# Advancements and Strategies for Selectivity Enhancement in Chemiresistive Gas Sensors

**DOI:** 10.3390/nano15171381

**Published:** 2025-09-08

**Authors:** Jianwei Liu, Jingyun Sun, Lei Zhu, Jiaxin Zhang, Xiaomeng Yang, Yating Zhang, Wei Yan

**Affiliations:** 1 School of Chemistry and Chemical Engineering, Xi’an University of Science & Technology, Xi’an 710054, China; liujianwei@xust.edu.cn (J.L.);; 2 Xi’an Key Laboratory of Solid Waste Resource Regeneration and Recycling, State Key Laboratory of Multiphase Flow Engineering, School of Energy and Power Engineering, Xi’an Jiaotong University, Xi’an 710049, China

**Keywords:** chemiresistive gas sensors, selectivity, semiconductor metal oxides, heterostructures, artificial intelligence

## Abstract

Chemiresistive gas sensors are extensively employed in environmental monitoring, disease diagnostics, and industrial safety due to their high sensitivity, low cost, and miniaturization. However, the high cross-sensitivity and poor selectivity of gas sensors limit their practical applications in complex environmental detection. In particular, the mechanisms underlying the selective response of certain chemiresistive materials to specific gases are not yet fully understood. In this review, we systematically discuss material design strategies and system integration techniques for enhancing the selectivity and sensitivity of gas sensors. The focus of material design primarily on the modification and optimization of advanced functional materials, including semiconductor metal oxides (SMOs), metallic/alloy systems, conjugated polymers (CPs), and two-dimensional nanomaterials. This study offers a comprehensive investigation into the underlying mechanisms for enhancing the gas sensing performance through oxygen vacancy modulation, single-atom catalysis, and heterojunction engineering. Furthermore, we explore the potential of emerging technologies, such as bionics and artificial intelligence, to synergistically integrate with functional sensitive materials, thereby achieving a significant enhancement in the selectivity of gas sensors. This review concludes by offering recommendations aimed at improving the selectivity of gas sensors, along with suggesting potential avenues for future research and development.

## 1. Introduction

Gas sensors are essential for various applications including environmental monitoring, industrial safety, disease diagnosis, smart agriculture and food quality assessment [1,2,3,4].

A variety of gas sensor technologies, each based on distinct operating principles, have been developed, including electrochemical, optical, catalytic combustion, and chemiresistive gas sensors [5]. Compared to these methods, chemiresistive sensors exhibit compelling advantages crucial for sensing applications. Electrochemical sensors offer good selectivity and sensitivity, but they often involve higher material costs and complex packaging [6,7]. Optical sensors provide high accuracy and stability but are typically bulky, expensive due to sophisticated light sources/detectors, and less amenable to miniaturization [8]. Catalytic combustion sensors are robust and simple but consume significant power, pose potential safety risks in explosive environments, and have limited scope for miniaturization [9]. In contrast, chemiresistive sensors stand out due to their significantly lower fabrication and operational costs, high sensitivity achievable through nanostructured materials, and inherent potential for miniaturization and integration into compact systems like portable devices and IoT nodes [10,11].

However, chemiresistive gas sensors often struggle to selectively detect and measure target gases in complex environments with multiple gases due to cross-sensitivity and poor selectivity. Therefore, the development of gas sensors with high sensitivity, excellent selectivity, and reliability is necessary for practical applications. The key to solving this problem lies in gaining an in-depth understanding of the strategies and mechanisms for enhancing gas sensing performance [12].

**Figure 1:**
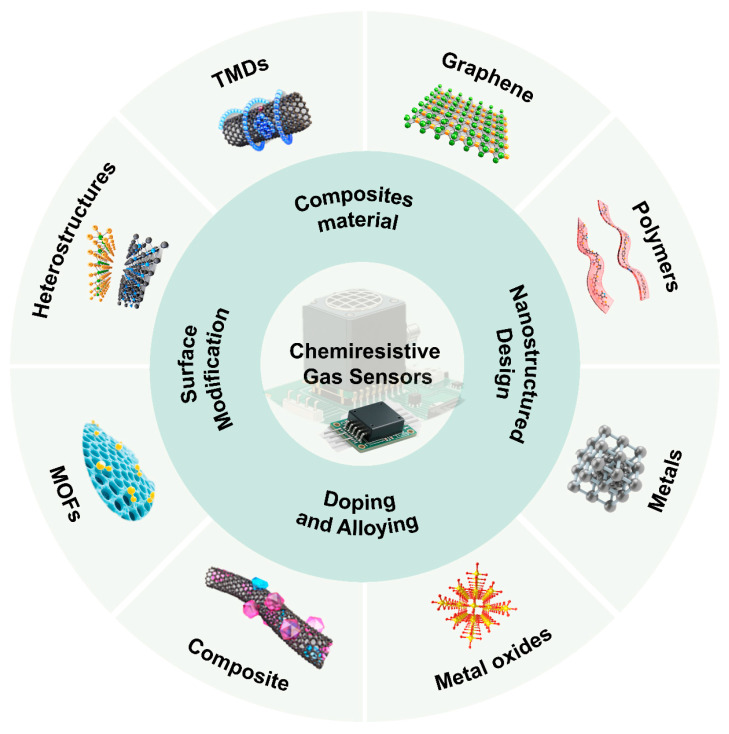
Schematic illustration of sensitive materials for chemiresistive gas sensors, and strategies for improving the gas sensing performance of sensors.

The working principle of chemiresistive gas sensors is based on the change in electrical conductivity of the sensitive material caused by redox reactions when exposed to the target gas environment. There are four main mechanisms based on different sensing material types (Figure 1). Among them, gas sensors based on semiconductor metal oxides (SMOs) are widely used due to their remarkable resistive modulation effect and well-established fabrication processes. To overcome the limitations of its intrinsic selectivity and relatively high operating temperature, researchers have developed a variety of refined modulation strategies, including noble metal nanoparticle catalysis [13], oxygen vacancy defect engineering [14] and heterojunction construction [15], aiming to optimize surface reaction activity and electron transport pathways [16]. Meanwhile, metal and alloy nanostructures, with their unique plasmonic effects and catalytic properties, demonstrate advantages in the detection of specific gases (such as H_2_) [17,18]. Conjugated polymers (CPs), with their solution processability, potential for room-temperature operation, and ease of molecular structure modification to achieve “lock-and-key” type recognition, offer new insights for flexible and wearable sensors [19]. Finally, two-dimensional materials represented by transition metal dichalcogenides (TMDs), graphene, and their derivatives, with their large specific surface area, high carrier mobility, and tunable electronic band structures, have paved the way for the development of highly sensitive and low-power devices [20,21,22].

With continuous advancements in materials science, the convergence of data science and system integration technologies is driving the emergence of transformative innovations in the field of chemiresistive sensors [23]. In addition, the synergistic integration of bionic technology and machine learning techniques offers a robust solution to the persistent challenge of gas identification within complex background environments [24]. Furthermore, there is significant variation in the methods used to evaluate gas selectivity across studies, and a standardized approach for assessing gas selectivity is lacking. Existing indicators based on response ratios struggle to reflect interference effects in real-world complex environments such as humidity, temperature fluctuations, and mixed gases resulting in poor comparability between studies. Therefore, the establishment of a standardized testing system is needed [25].

While extensive reviews have been documented on chemiresistive gas sensors [26,27], there has been limited comprehensive discussion on selectivity—a critical parameter that directly dictates the accuracy and reliability of sensing data in real-world applications. This review systematically elaborates on innovative strategies to overcome the poor selectivity of chemiresistive sensors. We emphasize material-level modifications, including crystal structure engineering, defect regulation (e.g., oxygen vacancy creation, doping), surface functionalization (e.g., noble metal decoration), and heterostructure construction. In addition, we introduce integration systems such as sensor arrays, and multi-modal sensing platforms for the practical implications. It also explores the current status, potential, and challenges of emerging disruptive technologies.

## 2. Gas Sensing Mechanism of Chemiresistive Gas Sensors

The gas sensing mechanism of chemiresistive gas sensors lies in the changes in the electrical conductivity due to the interactions between sensitive materials and target gas species. This involves three primary principles of a sensor: the receptor function (interaction with gas), the transducer function (chemical to electrical signal conversion), and the utility factor (gas diffusion efficiency) [28].

(1) Gas sensors based on semiconductor metal oxides

For typical semiconductor metal oxides, the sensing mechanism relies on oxygen species adsorption and redox reactions with the gas molecules. Initially, oxygen molecules adsorb onto the surface of semiconductor metal oxides (MOSs) and subsequently convert into various adsorbed oxygen species depending on the operating temperature. Below 150 °C, $O_{2\left(ads\right)}^{-}$ species exist as stable oxygen ions. Between 150 °C and 450 °C, $O_{\left(ads\right)}^{-}$ is stable. While above 450 °C, $O_{\left(ads\right)}^{2-}$ ions are the predominant species, as shown in the following Equations (1)–(4). This electron capture depletes near-surface carriers, increasing resistance. Reducing analyte gases react with these adsorbed oxygen species, releasing electrons and decreasing resistance.

(1) $$O_{2\left(gas\right)}\;\to \;O_{2\left(ads\right)}$$

(2) $$O_{2\left(gas\right)}+e^{-}\to O_{2\left(ads\right)}^{-}\;\left(T<150\;{}^{\circ}C\right)$$

(3) $$O_{2\left(ads\right)}^{-}+e^{-}\to 2O_{\left(ads\right)}^{-}\;\left(150\;{}^{\circ}C<T<450\;{}^{\circ}C\right)$$

(4) $$O_{2\left(ads\right)}+4e^{-}\to 2O_{\left(ads\right)}^{2-}\;\left(450\;{}^{\circ}C<T\right)$$

**Figure 2:**
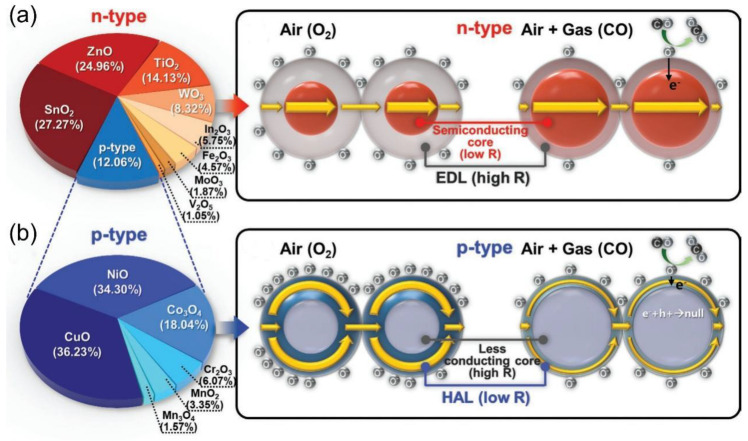
Gas sensing mechanisms of sensors based on (**a**) n-type and (**b**) p-type oxide semiconductor. Reproduced with permission from ref. [31]. Copyright 2020 John Wiley and Sons.

For n-type SMOs (e.g., ZnO, In_2_O_3_) [29,30], an electron depletion layer (EDL) forms due to surface oxygen adsorption and ionization, creating a “semiconductor core-high-resistance EDL shell” structure that requires overcoming a potential barrier for carrier transport. As shown in Figure 2a, when reducing gases such as CO contact the surface, they react with adsorbed oxygen, releasing electrons back into the EDL, which lowers its resistance and significantly decreases the sensor’s overall resistance [31]. For p-type semiconductor oxides (e.g., CuO and NiO) [32], surface oxygen adsorption captures electrons, leading to hole accumulation at the surface and forming a low-conductivity core with a hole accumulation layer (HAL) shell. This creates a dual-channel conduction system: a high-mobility HAL channel and a low-conductivity core channel. When reducing gases react, injected electrons recombine with HAL holes, reducing HAL conductivity and increasing the sensor’s overall resistance (Figure 2b) [31].

The gas sensitivity of SMOs is influenced by band structure, surface defects, grain size, chemical modifications, temperature, and humidity [33]. The semiconductor type (n-type or p-type) affects carrier transport direction, while micro/nano structures impact gas adsorption and reaction kinetics, affecting sensitivity, selectivity, and stability [26].

(2) Gas sensors based on two-dimensional materials

**Figure 3:**
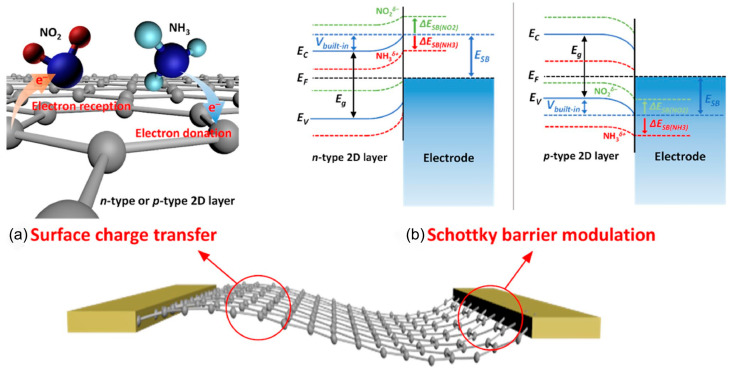
Schematic illustration of gas sensing mechanisms of sensors based on 2D layered nanomaterials (**a**) Surface charge transfer. (**b**) Schottky barrier modulation. Reproduced with permission from ref. [38]. Copyright 2018 Springer Nature.

The two-dimensional (2D) materials, such as graphene and TMDs, rely on direct charge transfer with gas molecules for sensing at room temperature [34,35,36], as displayed in Figure 3a. For n-type 2D semiconductors (e.g., MoS_2_ and MoSe_2_) [37], electron-accepting gases reduce electron concentration and increase resistance. For p-type 2D semiconductors (e.g., WSe_2_ and black phosphorus), electron acceptor gases increase hole concentration and decrease resistance, while electron donor gases reduce hole concentration and increase resistance. Gas adsorption not only causes band bending (ΔE_g_) and Fermi level (E_F_) shifts through surface charge transfer but also alters the Schottky barrier height (V_built-in_) at the material-metal electrode interface, impacting the transport efficiency of majority carriers and increasing resistance (Figure 3b) [38]. This dual mechanism, involving surface charge transfer and Schottky barrier modulation, underpins the high-sensitivity and reversible gas sensing capabilities of two-dimensional materials [39].

## 3. Selectivity Enhancement Strategies for Chemiresistive Gas Sensors

The performance of chemiresistive gas sensors is critically evaluated through several key parameters, including sensitivity, selectivity, long-term stability, response time, and the recovery characteristics signifying the device’s ability to return to its baseline resistance upon removal of the target gas [40]. The reversible response, underpinned by the efficient desorption of analyte molecules from the sensing material’s surface, is fundamental for the sensor’s practical utility and long-term reliability, enabling repeated and consistent measurements [41]. However, achieving high selectivity—the capability to distinguish a specific target gas accurately amidst complex mixtures containing potential interferents—remains one of the most persistent challenges for chemiresistive sensors. Overcoming this limitation necessitates deliberate modifications to the sensing materials or device architecture. Consequently, this chapter focuses on exploring various enhancement strategies specifically designed to augment the selectivity of chemiresistive gas sensors.

### 3.1. Semiconductor Metal Oxide-Based Gas Sensors

Gas sensors based on metal oxide semiconductor (MOS) primarily utilize n-type (e.g., ZnO, SnO_2_) and p-type (e.g., NiO, CuO) as sensing materials, which detect gases through electrical resistance modulation upon surface adsorption [16]. There are various approaches to significantly improve the selectivity of gas sensors based on SMOs. We present a discussion in the following two aspects: (1) functionalization of SMOs with catalysts, and (2) defect introduction and phase control. Catalytic functionalization enhances reactivity by depositing noble metals that dissociate oxygen molecules into reactive intermediates, accelerating target-specific gas reactions [13]. Defect generation and phase control modify material structures to generate oxygen vacancies or lattice defects, augmenting charge carrier density and creating preferential adsorption sites [14]. These methods collectively optimize surface chemistry and electronic properties for selective gas identification.

#### 3.1.1. Functionalization of Semiconductor Metal Oxides with Catalysts

Metal nanoparticles (NPs) demonstrate remarkable electronic properties and catalytic activity toward various gases [42,43,44]. The sensing performance of sensors is significantly enhanced by incorporating metal nanoparticles on the surface of SMOs. This is mainly attributed to the “chemical sensitization” achieved through the spillover effect of metal nanoparticles and the “electronic sensitization” resulting from the formation of metal–semiconductor Schottky contacts [45]. Particularly, noble metals such as platinum (Pt), palladium (Pd), gold (Au), and silver (Ag) are commonly used in catalysts to activate or dissociate gas molecules, accelerating their reactions with oxygen ions on the surface of SMOs [13]. Schottky barriers between these metals and SMOs can be adjusted by gas adsorption, thus affecting the resistance of the sensor.

**Figure 4:**
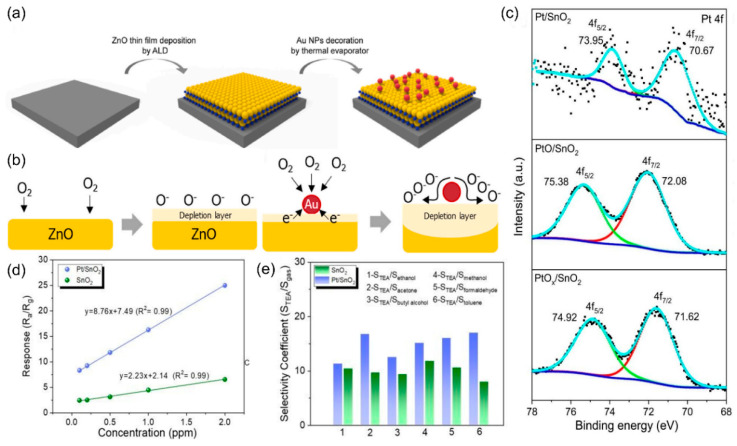
(**a**) Schematic illustration of the surface design of ZnO nanofilms with the strategic incorporation of Au NPs. (**b**) Schematic representation of the distinct sensing mechanisms for ZnO based gas sensors and Au NPs-decorated ZnO-based gas sensors. Reproduced with permission from ref. [48]. Copyright 2022 Elsevier. (**c**) The XPS spectra of Pt 4f in Pt/SnO_2_ thin films after annealing in Ar/H_2_ at 500 °C, PtO/SnO_2_ after annealing in air at 500 °C and as-deposited PtOx/SnO_2_ without annealing. (**d**) Linear fitting responses of SnO_2_ and Pt/SnO_2_ thin films. (**e**) Selectivity coefficients of SnO_2_ and Pt/SnO_2_ sensors toward various 10 ppm gases relative to TEA. Reproduced with permission from ref. [55]. Copyright 2020 Royal Society of Chemistry.

In addition, metal catalysts enhance the selectivity of gas sensors by providing specific surface active sites that preferentially catalyze the adsorption and dissociation of target gas molecules while simultaneously suppressing the reactions of non-target gases through electronic or geometric effects. For example, Pd-specific dissociative adsorption of H_2_ forming PdH_x_ intermediates for highly selective H_2_ sensing [46], and gold (Au) is effective for oxidizing flammable gases detection [47]. Bae et al. [48] synthesized Au NPs-decorated ZnO composite by atomic layer deposition and thermal evaporation method for gas sensing (Figure 4a). By controlling the size and density of the Au NPs, the sensor based on Au-ZnO achieved a 4.99% response for 50 ppb methyl mercaptan, with a detection limit of 50 ppb, and high selectivity against H_2_S. The improved sensing performance of the Au-ZnO sensor is attributed to the Au NPs catalyst, which enhances oxygen ionization, accelerates the oxidation of CH_3_SH, and thus increases the conductivity of the sensor (Figure 4b). In addition, the Au-ZnO sensor exhibits high sensitivity even at 73% humidity, offering a new potential method for the early diagnosis of periodontal disease. Teng et al. [49] present a one-step electrospinning synthesis of mesoporous PdO-functionalized SnO_2_ composite nanotubes (SPCTs) for highly selective NO_2_ detection at room temperature. The optimized 3-SPCT sensor exhibits exceptional selectivity toward NO_2_ over interfering gases (CO, H_2_, CH_4_, H_2_S, NH_3_), attributed to synergistic effects from PdO functionalization and unique nanostructure. The sensor based on SPCTs achieves a high response value of 23.3 toward 100 ppm NO_2_ with a rapid response time (1.33 s) and ultra-low detection limit (10 ppb).

Apart from single metal catalysts, combining two metals as catalysts for gas sensors can not only produce “electronic effects” (where one metal alters the electronic state of the other) and “geometric effects” (the formation of new active sites at the interface), but also enable “series catalysis” (the sequential activation of reactants) [13,50]. This design improves selectivity for complex gases, reduces interference, and boosts catalytic stability through modulation of metal ratios or the formation of core–shell/alloy nanostructures. For example, Liu et al. [51] studied AuPd alloys deposited on SnO_2_ hollow spheres for gas sensors. The AuPd-SnO_2_ sensor enables ultra-selective dimethyl disulfide (DMDS) detection via sulfur spillover. Charge transfer from AuPd to SnO_2_ strengthens DMDS adsorption (~2.28 eV), which is further confirmed by DFT. In situ DRIFT and XPS measurements reveal that DMDS can be decomposed to atomic S on AuPd and migrated to the surface of SnO_2_, reacting with adsorbed oxygen to release electrons. This pathway grants 36.6 response to 10 ppm DMDS at 135 °C. Moreover, due to the weaker adsorption and distinct kinetic behavior of interfering gases (such as hydrogen sulfide and acetone), the sensor achieves highly selective detection of DMDS. Le et al. [52] developed PdAu alloy encapsulated within ZnO shells. The synergistic effects of the composite structure enable Au to enrich surface oxygen species, while Pd in the alloy resists oxidation, thereby preserving hydrogen adsorption sites. The PdAu-ZnO sensors yield a high response value of 80 toward 100 ppm H_2_ at 300 °C and exceptional selectivity. Li et al. [53] functionalized PdPt bimetallic nanoparticles with a Pd-rich shell and Pt-rich core on SnO_2_ nanosheets, achieving temperature-dependent dual selectivity: high response to CO at 100 °C and the ability to detect CH_4_ at 320 °C. This enhanced selectivity is attributed to synergistic chemical sensitization, PdO-mediated electronic sensitization, and Schottky barrier modulation at the metal-semiconductor interface.

To further improve the utilization efficiency of metal catalysts, single-atom catalysts (SACs) have been proposed for gas sensors [54]. This sensing material design is composed of atomically isolated catalyst atoms stabilized on the surface of the support SMOs materials. SACs improve catalytic efficiency through chemical sensitization. A study demonstrated that using atomic layer deposition (ALD) to anchor platinum (Pt) atoms on a 9 nm tin dioxide (SnO_2_) film forms a Pt-SnO_2_ SAC system [55]. High-angle annular dark-field scanning transmission electron microscopy confirmed the uniform dispersion of Pt atoms, while X-ray photoelectron spectroscopy showed Pt in a metallic state (Pt 0) with strong bonding to SnO_2_ (Figure 4c). The sensor based on Pt-SnO_2_ exhibits exceptional sensing performance toward triethylamine (TEA) with a response value of 136.2 at 200 °C, 9 times higher than that of pure SnO_2_ (8.76), as shown in Figure 4d. In addition, the sensor shows low detection limit of 7 ppb, and rapid response/recovery times of 3 and 6 s. As shown in Figure 4e, compared with pristine SnO_2_ sensor, the Pt-SnO_2_ sensor exhibited significantly enhanced selectivity toward TEA detection, demonstrating that the single-atom Pt catalysts stabilized on the surface of SnO_2_ serve as favorable adsorption sites for TEA molecules over other interferent gas species.

#### 3.1.2. Defects Generation and Phase Control

**Figure 5:**
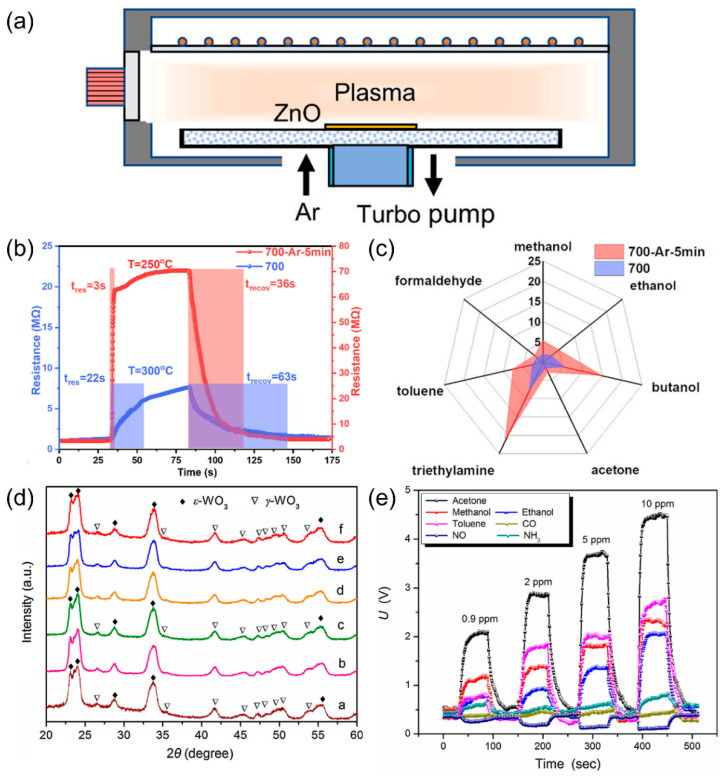
(**a**) Ar plasma treatment of ZnO films. (**b**) Response recovery time of ZnO sensors to 10 ppm TEA at optimal operating temperature before and after Ar plasma treatment. (**c**) Responses of ZnO film sensors before and after 5 min Ar plasma treatment to 10 ppm gases. Reproduced with permission from ref. [60]. Copyright 2021 Elsevier. (**d**) XRD patterns of W_0_ a, FW_1_ b, FW_2_ c, FW_3_ d, FW_4_ e and FW_5_ f. (**e**) Dynamic curves of FW_3_ sensor to acetone, ethanol, methanol, toluene, NH_3_, CO, and NO. Reproduced with permission from ref. [64]. Copyright 2018 Elsevier.

Experimental evidence proves that defect engineering in SMOs sensing materials plays an essential role in tuning the electronic structure and electrical conductivity of SMOs, as well as enhancing the number of active sites for chemical reactions [14]. These combined effects contribute to improved gas adsorption characteristics. In the field of defect engineering, methods such as ultraviolet irradiation [56,57], defect induction [58], plasma etching [59,60], and ion irradiation [61] have been demonstrated to introduce defects and reduce the coordination number of surface atoms. In particular, the introduction of controllable oxygen vacancies can narrow the band gap, increase the carrier concentration, and enhance the chemical adsorption of target gases [14]. The literature reports that the selectivity of ZnO-based gas sensors can be effectively enhanced via defect engineering. Li et al. [60] introduced oxygen vacancies into ultrathin ZnO films using Ar plasma (Figure 5a). The gas sensor exhibited a significantly enhanced selectivity toward triethylamine (TEA) (Figure 5b,c). This improvement is attributed to the relatively low C-N bond energy of TEA (307 kJ/mol), which makes it more likely to react at oxygen vacancy sites. Shin et al. [61] irradiated ZnO nanoparticles with Xe ions and achieved the optimization of oxygen vacancies at a dose of 1 × 10^15^ ions/cm^2^. The sensor achieves a high response value of 88.5 toward 10 ppm NO_2_ and excellent selectivity. This is mainly attributed to the high electron affinity (2.28 eV) and low O-NO bond energy (305 kJ/mol) of NO_2_, which makes it more prone to adsorption on vacancy-rich surfaces. The above two methods both achieve highly selective responses to specific gases through defective engineering. Paolucci et al. [62] controlled oxidation of SnSe_2_ yields amorphous 2D a-SnO_2_ interfaces with exceptional stability. DFT reveals competitive dissociative chemisorption of H_2_S and H_2_O at identical sites, explaining humidity-induced H_2_S signal reduction (RR = 2.4 → 1.9 at 1 ppm) and LOD increase (210 → 380 ppb), enabling selective H_2_S detection amid humidity interference.

While defects provide active sites for target gas molecules-thereby improving sensitivity and response speed-they can also disrupt the material’s electronic structure, leading to increased noise, baseline drift, and reduced carrier mobility. For instance, ZnO nanorod arrays with moderate oxygen vacancies exhibit superior ethanol sensitivity due to tailored carrier behavior, whereas excessive vacancies induce unstable metal-semiconductor contacts and non-linear I-V characteristics, compromising accuracy [63]. Additionally, excessive defect generation may introduce charge carrier recombination centers, impairing carrier mobility and reducing conductivity. Thus, defect optimization—rather than maximization—is essential to harness beneficial sites while preserving the sensing matrix’s coherence and reliability.

Besides defect generation, phase control is another key strategy to realize high sensitivity and selectivity of gas sensors based on SMOs. Shen et al. [64] investigated Fe-C codoped ε-phase WO_3_ for acetone sensing. The ε-phase of WO_3_ is stabilized through doping with chromium (Cr) or silicon (Si). The XRD patterns in Figure 5d confirm the ε-phase of WO_3_. The selectivity of the ε-phase WO_3_ originates from its non-centrosymmetric crystal structure. This structure generates a spontaneous dipole moment, which attracts polar acetone molecules and promotes their oxidation. As shown in Figure 5e, the FW_3_ sensor shows high sensitivity of 7.3 toward 0.9 ppm acetone as well as high selectivity.

### 3.2. Pure Metal and Alloy-Based Gas Sensors

The enhancement of selectivity in pure metal and alloy-based chemiresistive gas sensors primarily relies on the distinct electronic and catalytic properties of these materials upon interaction with target gases. Noble metal-based sensors, utilizing elements such as platinum and palladium, exhibit exceptional sensitivity and selectivity due to their high catalytic activity to dissociate gas molecules [65]. Meanwhile, metal alloy-based sensors benefit from synergistic effects and tailored electronic structures, enabling improved selectivity through composition optimization. This section is crucial as it highlights strategies for designing highly selective sensing interfaces without employing complex composite structures, thereby offering fundamental insights into material–gas interactions and guiding the development of efficient and stable gas sensors.

#### 3.2.1. Noble Metal-Based Sensors

**Figure 6:**
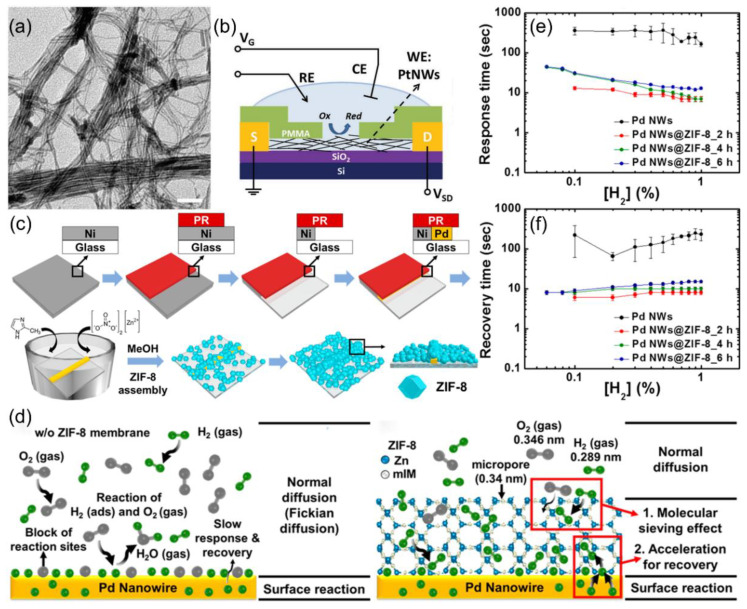
(**a**) TEM image of as-prepared ultrathin PtNWs with a diameter of ≈3 nm. (**b**) Schematic illustration of the electrochemical cleaning set up for the PtNW sensor device. Reproduced with permission from ref. [69]. Copyright 2016 John Wiley and Sons. (**c**) Schematic illustration of the synthesis of Pd NWs@ZIF-8 using the LPNE process and the following assembly process. (**d**) Schematics of the sensing model for Pd NWs without and with ZIF-8 membrane. The ZIF-8 (0.34 nm in size) membrane filters out O_2_ (0.346 nm), allowing only H_2_ (0.29 nm) to reach the Pd NWs. (**e**) Response times, and (**f**) recovery times of Pd NWs and Pd NWs/ZIF-8 sensors against H_2_ concentrations in air. Various thicknesses of ZIF-8 onto Pd NWs were achieved by varying the assembly time at 2, 4, and 6 h. Reproduced with permission from ref. [70]. Copyright 2017 American Chemical Society.

Noble metals such as platinum (Pt) [66] and palladium (Pd) [67] are sensitive to hydrogen and carbon monoxide due to their catalytic properties. However, the selectivity of pure metals is generally limited because their surface-active sites are relatively uniform and prone to cross-interference from various gases with similar chemical properties. Recently, ultra-fine metal nanowires (with diameters < 5 nm) have become a research hotspot due to their extremely high specific surface area and quantum confinement effects [68]. Ultra-fine platinum nanowires enhance gas adsorption kinetics by regulating surface states, emphasizing the importance of metal properties in achieving selectivity. Ding et al. [69] synthesized ligand-free ultra-fine Pt nanowires (~3 nm diameter, shown in Figure 6a) for H_2_ sensing. The gas sensor based on platinum nanowires achieves high sensitivity with response of 261.4% toward 0.5% H_2_, which increased to 768% with alkylamine modification. Their selectivity is due to enhanced surface scattering from Pt-H bonding, while thiol/amine modifications control gas diffusion and reduce humidity interference. Figure 6b illustrates the electrochemical cleaning setup, which removes impurities to achieve reversible response, solving a key issue for wet-synthesized sensors.

Research on Pd nanowires addresses the issue of oxygen interference in air. Koo et al. [70] introduced ZIF-8 metal–organic frameworks as a nanofiltration layer with 0.34 nm micropores for molecular sieving (Figure 6c). The nanofiltration layer allows H_2_ with the kinetic diameter of 0.289 nm to pass while blocking O_2_ with the kinetic diameter of 0.345 nm, achieving effective H_2_ gas sieving, as shown in Figure 6d. This innovation improved sensor response and recovery speed by 20 times (7 s to respond, 10 s to recover at 1% H_2_), despite a slight decrease from 5.9% to 3.5% in response, as shown in Figure 6e,f. The detection limit improved to 600 ppm, demonstrating the benefits of combining physical filtration and chemical sensing. Pure metal chemical resistance sensors enhance sensitivity and selectivity by controlling surface defects and phase interfaces, offering a reliable solution for hydrogen sensors in complex environments.

#### 3.2.2. Metal Alloy-Based Sensors

**Figure 7:**
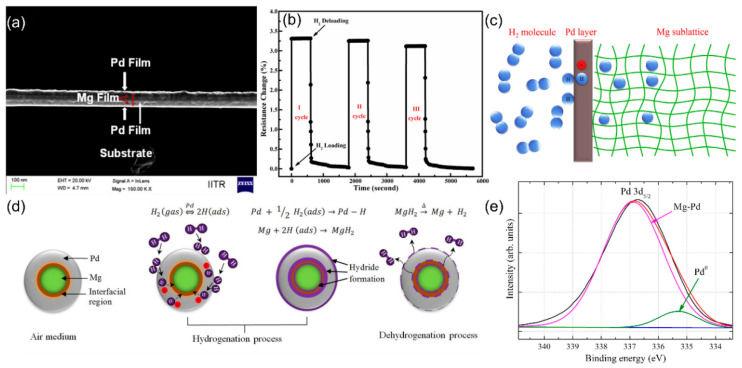
(**a**) FE-SEM cross-section images of as-deposited of Pd/Mg/Pd/Mg/Pd multilayers. (**b**) Pd/Mg thin films, during hydrogenation (2 bar H_2_) and dehydrogenation at different temperatures. (**c**) Schematic diagram of reaction mechanism between hydrogen and Pd/Mg lattice. Reproduced with permission from ref. [71]. Copyright 2015 Elsevier. (**d**) Schematic of the hydrogen sensing mechanism for Pd-capped Mg bimetallic NPs. (**e**) XPS spectra of Pd5/2 electrons. Reproduced with permission from ref. [72]. Copyright 2017 Elsevier.

Metal alloy-based sensors have been developed to overcome the limitations of pure metals and further improve the selectivity. Alloying multiple metals enables precise tuning of the electronic structure, surface energy, and catalytic properties, resulting in significant synergistic effects for gas sensors. Several studies reported that alloy/bimetallic structures (e.g., Pd-Mg [71,72], Pd-Y [73], Pd-Pt [74]) can optimize the sensitivity of chemiresistive gas sensors. By employing nanostructured designs, they address the issues of expansion, oxidation, and stability commonly found in traditional Pd-based sensors, thereby achieving highly sensitive and fast-response room-temperature hydrogen detection. For instance, Gautam et al. [71] used magnetron sputtering technology to fabricate Pd-Mg alloys and multilayer films structures (Figure 7a). The sensor based on Pd-Mg alloys achieves a rapid and reversible response in 3 s at room temperature (ΔR = 3.3% @ 2 bar H_2_) (Figure 7b). This is because palladium (Pd) doping reduces the hydrogen binding energy in the magnesium lattice, thereby enhancing hydrogen desorption at the Pd/Mg interface (Figure 7c). Hassan et al. [72] investigated quantum-sized Pd/Mg bimetallic films (~6 nm) on a hydrophobic Al_2_O_3_ substrate for H_2_ sensor. By exploiting nanoparticle-induced lattice expansion to modulate conductivity, this approach not only achieved ultrafast response and wide-range hydrogen detection (1–40,000 ppm), but also effectively suppressed humidity interference through the hydrophobic interface. As shown in Figure 7d,e, XPS and COMSOL simulations highlighted charge transfer at the Pd-Mg interface, supporting Gautam’s synergistic mechanism.

Furthermore, Wang et al. [73] proposed a Pd-Y (Pd_0.92_Y_0.08_) alloy for H_2_ gas sensor, where Y doping expands the Pd lattice spacing, effectively preventing hydrogen-induced cracking while enhancing sensitivity (29.28% at 4% H_2_). The ultra-thin structure significantly accelerates response time (83 s at 4% H_2_) by shortening the hydrogen diffusion path. The nanocrystalline Pd (<10 nm) exhibits a gap effect that allows switch-type sensing. Moreover, Kim et al. [74] deposited a Pd@Pt layer on polyacrylonitrile nanofiber yarns, revealing that resistive switching behavior depends on hydrogen concentration: at high levels (>1%), electron scattering is dominant (ΔR/R_0_ > 0), while at low levels (<0.5%), lattice expansion reduces the gap (ΔR/R_0_ < 0). The core–shell structure also mitigates Pd volume expansion. These findings suggest that alloy-based chemiresistive sensors show great potential for enhanced selectivity and sensitivity.

### 3.3. Conjugated Polymer-Based Gas Sensors

The enhancement of selectivity in conjugated polymer-based gas sensors primarily revolves around strategic molecular engineering approaches, notably through doping and functionalization as well as composite structure design. Doping with oxidizing or reducing agents and introducing functional side groups can significantly modulate the electron density and binding affinity of polymer chains, thereby improving specificity toward target gases [75]. Meanwhile, compositing with inorganic nanoparticles or carbon nanomaterials further enhances selectivity via synergistic effects and interface-dominated sensing mechanisms. This section will focus on recent advances in these two strategic directions for achieving highly selective chemiresistive sensing using conjugated polymers.

#### 3.3.1. Doping and Functionalization

Conjugated polymers (CPs) are ideal for gas sensing due to their customizable π-conjugated backbone, semiconductor properties, and structural flexibility. However, their practical applications are limited by the following factors: weak signals caused by inherently low electrical conductivity, a lack of selective recognition capability for different gases, and poor long-term stability. The strategies of doping and functionalization of conjugated polymers have been widely adopted to overcome the above issues [76,77]. Doping improves conductivity by adjusting the polymer’s energy levels and carrier concentration. For instance, Ngai et al. [78] developed a donor-acceptor polymer, PDEB, which transforms into PDNB at 200 °C, enhancing solvent resistance. PDNB’s narrow bandgap and high HOMO level allow effective p-type doping with HCl, resulting in a stable, conductive material (PDNB: HCl) with a conductivity of 0.24 S cm^−1^. The sensor based on PDNB can identify 10 different types of volatile organic liquids at a low voltage of 1 V, with each liquid producing a unique current-time curve. Moreover, the sensor is reusable for hundreds of cycles. This study provides a high-performance material platform for reusable chemiresistive sensors.

**Figure 8:**
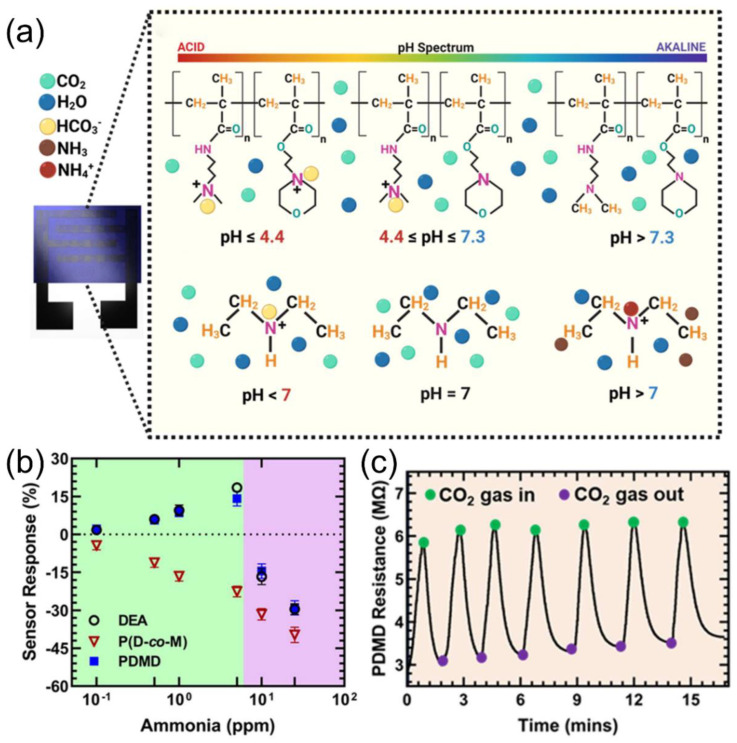
(**a**) P (D-co-M) protonation with carbonic acid ion over a wide pH range (4.4–7.3) and DEA protonation in the presence of carbonic acid ion and ammonium ion. (**b**) PDMD, DEA, and P (D-co-M) solid-state sensor response to NH_3_ gas (0.1–25 ppm). (**c**) PDMD sensor recovery and reversibility in the presence and absence of humidified CO_2_ gas. Reproduced with permission from ref. [79]. Copyright 2024 American Chemical Society.

To further address the issue of cross-sensitivity in complex environments, specific functional groups can be introduced into the polymer backbone or side chains. This enables the polymer to engage in specific interactions with gas molecules, thereby distinguishing the target gas in complex environments and enhancing the selectivity of gas sensors. Rath et al. [79] introduced diethylamine (DEA) into the CO_2_-responsive copolymer P (D-co-M), where amine groups gradually protonate under CO_2_, increasing ionic conductivity as pH drops (pH 4.4–7.3), as displayed in Figure 8a. The high pKa of DEA (10.64) enables the protonation of NH_3_ (pKa = 9.25), significantly reducing ammonia interference and achieving CO_2_/NH_3_ discrimination (Figure 8b). The sensor performs wide-range CO_2_ detection (10^3^–10^6^ ppm) in high-humidity environments (>80% RH) while substantially shortening the response time (Figure 8c). In another study, Rath et al. [80] demonstrated the effectiveness of functional group design for specific detection by developing a highly selective ammonia sensor using electrostatic interactions between polymers and NH_4_^+^. Yang et al. [81] incorporated polar triethylene glycol (TEG) side chains into a heat-resistant matrix for a DPP-based organic mixed conductor-polyimide blend film for enhancing NO_2_ selectivity and response. These studies demonstrate that systematic optimization of the conductivity, selectivity, and environmental tolerance of gas sensors can be achieved through backbone energy level engineering, side-chain polarity modification, and functional group regulation. This advancement promotes their application in complex scenarios such as industrial emissions and medical diagnostics.

#### 3.3.2. Composite Structure Design

Composite structure design enhances performance by utilizing the synergistic effects of various materials through hybrid systems. This involves three main strategies: carbon-based hybridization, metal hybridization, and porous framework hybridization. For instance, combining carbon materials such as carbon nanotubes and graphene improves sensor sensitivity due to their high conductivity and abundant adsorption sites, which facilitate selective gas molecule adsorption and optimize charge transport. He et al. [81] creation of the PSATT-7NC hybrid material by combining amino-functionalized multi-walled carbon nanotubes (NH_2_-MWCNTs) with conjugated microporous polymers (PSATT). This structure leverages the high electrical conductivity of NH_2_-MWCNTs for electronic transport and enhances charge transfer through π-π interactions, achieving high response of 9766% toward 4 ppm NO_2_, which is 2.5 times higher than that of pure PSATT and a low detection limit of 0.79 ppb at 100 °C, as well as excellent selectivity and stability.

In addition, incorporating noble metal nanoparticles or metal oxides such as gold, silver, platinum, and palladium into the polymer matrix enables highly selective and sensitive detection of specific reactive gases or biomolecules. Thangamani et al. [82] enhanced the polypyrrole/polyvinyl alcohol (PVA/WPPy) matrix with V_2_O_5_ nanorods to create flexible nanocomposites. These composites show strong ionic dipole interactions, enabling reversible LPG adsorption at room temperature. With 15 wt.% filler, the material achieves 1.16% response to 600 ppm LPG with a rapid response/recovery time of 10 s/8 s, and high selectivity over gases such as acetone and benzene.

**Figure 9:**
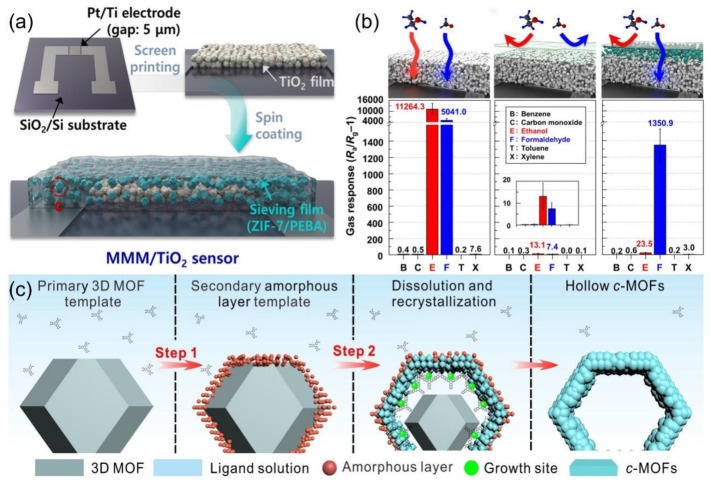
(**a**) Schematic illustration of steps for the fabrication of MMM coated TiO_2_ sensors. (**b**) Gas responses of a bare TiO_2_, Pure PEBA/TiO_2_, and 5MMM/TiO_2_ sensors exposed to 5 ppm gas at 23 °C under UV illumination (wavelength: 365 nm). Reproduced with permission from ref. [83]. Copyright 2021 Springer Nature. (**c**) Schematic overview of the two-stepped transformation mechanism of ZIF-8 NPs to hollow Zn-HHTP NPs. Reproduced with permission from ref. [84]. Copyright 2023 John Wiley and Sons.

The composite of conjugated polymers with metal–organic frameworks (MOFs) has been proven to improve the selectivity and sensitivity of sensors. These framework materials possess highly ordered pore structures, large surface areas, and abundant active sites, which modulate the resistance signals through interactions at the interface with the conjugated polymers. For example, Jo et al. [83] developed a composite structure using a mixed matrix membrane (MMM) for selective formaldehyde detection at room temperature. ZIF-7 nanoparticles (~139 nm) are dispersed in a PEBA polymer matrix to form an MMM coating on a TiO_2_ thin film in Figure 9a. The hexagonal channels of ZIF-7 (~0.3 nm) selectivity block interfering with gases such as ethanol through molecular sieving effects, while allowing formaldehyde molecules to permeate. Combined with the photoactivation properties of TiO_2_, the sensor enables specific detection of formaldehyde at concentrations as low as 25 ppb (Figure 9b). This design combines MOF/polymer membranes with photoactivated sensing, enhancing flexible device integration and addressing response intensity and mass transfer efficiency. Huang et al. [84] proposed a method for the conversion from insulation to conductive MOFs by creating 12 types of 2D c-MOFs with hierarchical nanostructures. As shown in Figure 9c, this work utilizes HKUST-1 and other 3D MbbOF as sacrificial templates, converting them into 2D conjugated MOFs with hollow nanostructures at room temperature. These materials exhibit both high crystallinity and large surface area, promoting exposure to the active site and gas diffusion, thereby making them ideal for the design of high-performance sensors.

### 3.4. Two-Dimensional Materials-Based Gas Sensors

This section delves into the enhanced selectivity of chemiresistive gas sensors utilizing two-dimensional materials, with a focus on transition metal dichalcogenides (TMDs) and graphene and its derivatives-based gas sensors. TMDs such as MoS_2_ exhibit tunable electronic properties and layer-dependent sensitivity, which facilitate selective gas detection via metal doping, and heterojunction construction [85]. Graphene and its derivatives, including graphene oxide and reduced graphene oxide, are widely modified through chemical functionalization, elemental doping, and hybridization with other nanomaterials to promote specific gas adsorption and discrimination [86]. Key strategies including heteroatom doping, composite formation, and surface modification will be discussed to illustrate how material tailoring significantly boosts selective detection capabilities.

#### 3.4.1. Transition Metal Dichalcogenides (TMDs)

Two-dimensional materials, with unique distinct physical and chemical properties, promise to construct high-performance chemiresistive sensors [87]. Transition metal dichalcogenides (TMDs, such as MoS_2_, WS_2_, and MoSe_2_), with their tunable band gaps and abundant edge-active sites, are particularly suited for gas sensors. Studies have confirmed that incorporating noble metal nanoparticles (e.g., Pt [36], Ni [88], Co [89]) into TMDs can improve the sensitivity and stability of sensors at room temperature by increasing the specific surface area, providing active sites, catalyzing gas dissociation, and modulating the interfacial band structures. Bharathi team [88] conducted studies highlighting how different transition metal dopants affect MoS_2_ nanostructures and enhance NO_2_ sensing. They synthesized nickel (Ni)-doped MoS_2_ nanosheets (with 3–7at.% Ni) via a hydrothermal method. This study revealed that Ni doping optimized material morphology, inducing the self-assembly of nanosheets into porous nanoflower structures and exposing more edge-active sites [88]. Among them, the sensor based on 7at.% Ni-MoS_2_ showed a response of 45.25% to 200 ppm NO_2_ and demonstrated long-term stability of 80.4% over 30 days. In addition, they further investigated the effect of cobalt (Co) doping and confirmed that 7 at.% cobalt doping in MoS_2_ yields optimal NO_2_ sensing performance due to maximized edge-active sites and vertically aligned nanosheet morphology, enhancing gas adsorption and charge transfer. Below 7 at.% (e.g., 3–5 at.%), insufficient defect sites limit response enhancement. The XPS measurements confirmed that 7 at.% cobalt doping in MoS_2_ induces a Fermi level shift, which also enhances conductivity. However, excessive cobalt doping exceeding 7 at.% disrupt the MoS_2_ lattice structure, compromising charge carrier mobility. Thus, an appropriate amount of 7 at.% cobalt doping in MoS_2_ not only maintains the structural integrity of the gas-sensitive material but also maximizes the number of active sites, thereby significantly enhancing the gas-sensing performance [89].

**Figure 10:**
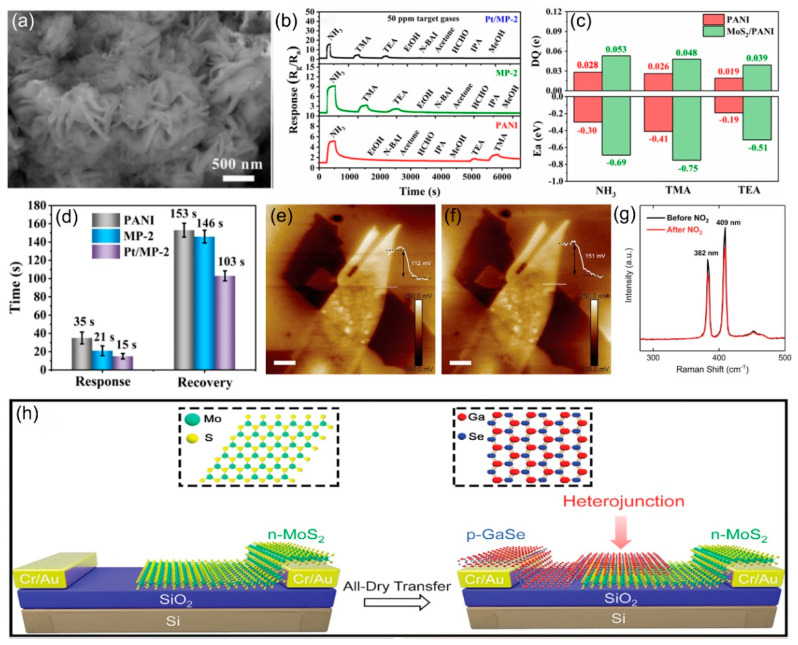
(**a**) SEM images of Pt/MoS_2_/PANI (6%, Pt/MP-2). (**b**) Gas-sensing responses of PANI, MP-2, and Pt/MP-2 flexible gas sensors to the concentration of 50 ppm of various gases at RT. (**c**) Adsorption energy values (E_a_) and Bader’s charge (ΔQ) of PANI and MoS_2_ (002)/PANI to NH_3_, TMA, and TEM gases. (**d**) Average response and recovery times of PANI, MP-2, and Pt/MP-2 flexible gas sensors to 50 ppm NH_3_ vapor at RT. Reproduced with permission from ref. [90]. Copyright 2023 American Chemical Society. KPFM images of the heterojunction (**e**) before and (**f**) after exposing to NO_2_. (**g**) Raman spectra of the heterojunction before and after NO_2_ exposure at room temperature. (**h**) Schematic diagram of the fabrication of the MoS2/GaSe heterojunction. Reproduced with permission from ref. [92]. Copyright 2021 John Wiley and Sons.

In contrast, Tian et al. [90] developed a Pt NPs-modified MoS_2_/polyaniline nanocomposite (Pt/MoS_2_/PANI) for room-temperature NH_3_ detection, as shown in Figure 10a. Figure 10b demonstrates the superior selectivity of PANI, MP-2, and Pt/MP-2 sensors toward NH_3_ over other VOC gases (including TMA and TEA) at 50 ppm, while Figure 10c reveals through DFT calculations that despite TMA exhibiting higher adsorption energy, NH_3_ facilitates the largest charge transfer (ΔQ) to both PANI and MoS_2_ (002)/PANI models, explaining the experimental selectivity. Pt/MoS_2_/PANI efficiently detects NH_3_ through p-n heterojunctions, Schottky barriers, and Pt’s catalytic effect, with a fast response/recovery time of 15 s/103 s (Figure 10d). Li et al. [91] fabricated Pt-modified MoSe_2_ for room-temperature NO_2_ detection. This study combines experiments and theory to explain the gas-sensing mechanism of metal-modified TMDs, where target gas adsorption alters carrier concentration and mobility, enhancing sensor selectivity through metal oxide composite heterostructures. Niu et al. [92] developed a groundbreaking photovoltaic self-powered gas sensor achieving outstanding NO_2_ detection at room temperature with a limit detection of 20 ppb and rapid response/recovery times of 23 s/178 s. As shown in Figure 10e–g, KPFM and Raman analysis showed that NO_2_ reduces the short-circuit current by capturing MoS_2_ conduction band electrons, causing Fermi level shifts. The vertically stacked MoS_2_/GaSe heterojunction was fabricated with all-dry transfer method as illustrated in Figure 10h. This study introduces GaSe into heterojunction sensing for the first time, providing a new paradigm for ultra-low power ppb-level detection.

#### 3.4.2. Graphene and Its Derivatives-Based Gas Sensors

Graphene, a single layer of carbon atoms in a two-dimensional honeycomb structure, is promising for sensing applications due to its large surface area and excellent conductivity [36,93,94]. However, graphene-based gas sensors respond to all gases and cannot achieve selective gas detection. Therefore, to enhance the sensing performance of graphene, its selectivity can be greatly improved through defect engineering, graphene oxide (GO) [66], reduced graphene oxide (rGO) [95], and functionalization can enhance selectivity. Eigler and Feicht et al. [96] discussed strategies for regulating defects in GO, suggesting that vacancy defects can serve as active sites for gas adsorption, while hole defects can regulate the molecular sieving effect. This research provides a theoretical basis for developing sensitive graphene-based gas sensors.

**Figure 11:**
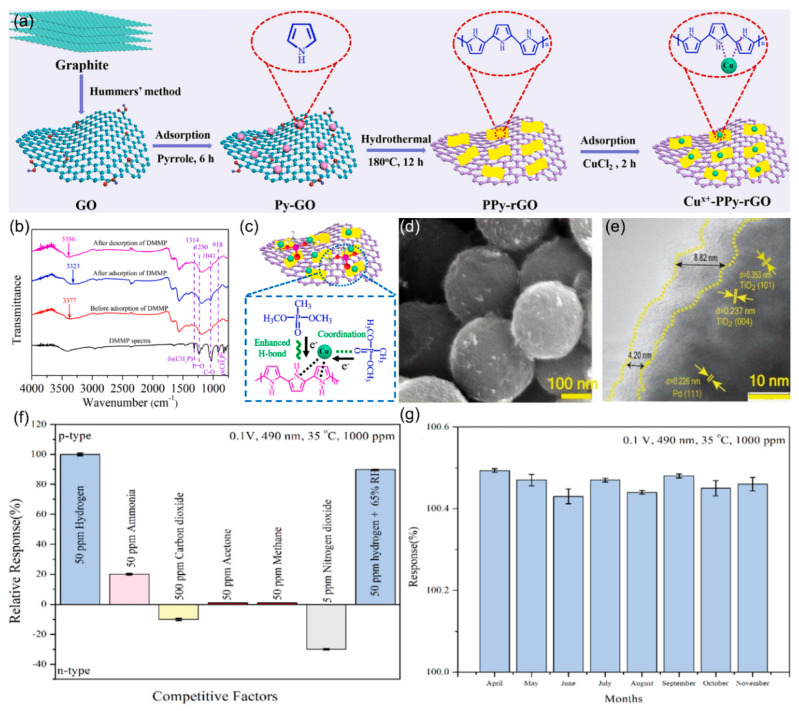
(**a**) Schematic illustration of the fabrication process of Cu^x+^-PPy-rGO hybrids. (**b**) FT-IR spectra of Cu^x+^-PPy-rGO sample before and after adsorbing DMMP. (**c**) Schematic illustration of the DMMP sensing mechanism on Cu^x+^-PPy-rGO hybrids. Reproduced with permission from ref. [98]. Copyright 2023 Elsevier. (**d**) SEM images of rGPT. (**e**) HR-TEM images for rGO encapsulated shell and crystalline planes of TiO_2_-NSand Pd-QDs. (**f**) Selectivity and (**g**) long term stability of the fabricated rGPT-NS at 35 °C under 0.1 V and 490 nm wavelength. Reproduced with permission from ref. [99]. Copyright 2023 Elsevier.

Furthermore, the practical application of graphene-based materials is often limited by issues such as poor interfacial contact and uneven thickness caused by insufficient precision in the assembly of nanostructures. Wang et al. [97] addressed this by using alternating current dielectrophoresis (DEP) with optimized parameters (500 kHz frequency, 10 V voltage, 30 s duration) for precise assembly of graphene oxide (GO) nanostructures. This method leverages the GO’s oxygen-containing groups for improved hydrogen gas selectivity and response. Additionally, atomic-level interface engineering can enhance the sensitivity of sensing materials. Yang et al. [98] developed a room-temperature DMMP sensor using polypyrrole-reduced graphene oxide (PPy-rGO) with isolated Cu-N_5_ sites in Figure 11a. These sites enhance hydrogen bonding and use unsaturated Cu^+^ as Lewis acid sites to adsorb DMMP’s oxygen atoms (Figure 11b,c). Therefore, the sensor’s response increased by 4.5 times, the detection limit decreased by 2.5 times, and the response/recovery times were 49 s/106 s.

Additionally, rGO-encapsulated photosensitive heterojunctions can enhance sensor selectivity by promoting charge separation. The Shafiei team [99] developed a composite system of rGO-encapsulated Pd quantum dots on TiO_2_ nanospheres (rGPT-NS)forming multiple heterojunctions that enhance hydrogen selectivity (Figure 11d,e). As quantified in Figure 11f, it exhibits 100% response to 50 ppm H_2_ while exhibiting minimal cross-sensitivity to competing gases (<1% for 50 ppm CH_4_/acetone), maintaining 99% ± 0.09 response under 65% humidity. Critically, Figure 11g demonstrates < 3% signal attenuation over 8 months toward 1000 ppm H_2_, confirming long-term selectivity. The superior selectivity is attributed to the synergistic effects of Pd’s catalytic specificity for hydrogen dissociation, rGO’s selective gas adsorption properties, and visible-light-activated surface reactions that preferentially modulate hydrogen interactions over competing gases.

More importantly, the surface of graphene allows for the covalent grafting of MOFs. Through size matching, electrostatic interactions, or specific chemical reactions, it enables highly selective recognition of target molecules. Jayaramulu et al. [100] developed GA@UiO-66-NH_2_ hybrids by covalently bonding graphene acid’s carboxyl groups with MOFs’ amino groups, ensuring stable chemical coupling. Graphene acts as both a charge transport network and enhances gas diffusion through its microporous-mesoporous structure. The sensor, leveraging amide bond and CO_2_ interactions, achieves a 10% response at 200 °C with response time of 18 s. In situ Raman spectroscopy first confirmed the reversible chemical adsorption mechanism.

Therefore, the key to enhancing the selectivity of two-dimensional material-based chemiresistive sensors lies in the precise regulation of the intrinsic electronic structure and surface chemical properties of the materials, as well as in the design and optimization of specific interactions between the sensing interface and the analytes. This approach is fundamental for advancing next-generation gas and chemiresistive sensors with excellent selectivity.

As displayed in Table 1, we summarize the effects of different modification methods on the selectivity and gas-sensing performance of chemiresistive gas sensors as reported in the literature.

## 4. Novel Selective and System Integration Strategies

### 4.1. Bionics and Heterogeneous Structures

Among the strategies for enhancing the selectivity of chemiresistive gas sensors, inspired by the biological olfactory system, researchers have sought to improve the recognition of specific target molecules. This is achieved by mimicking its highly specific receptor arrays and signal processing mechanisms, leveraging energy band modulation between different materials, interfacial charge transfer, and synergistic sensitization effects. Chun et al. [114] proposed a chemiresistive gas sensor based on oxygen vacancy dynamics (Pt/TiO_2_ nanorod/TiN structure), overcoming the limitations of traditional sensing mechanisms. This device uses redox reactions for quick response and recovery to reductive gases H_2_ at room temperature and features an intrinsic memory effect, allowing gas pulse counting and risk grading without external storage. By alternately introducing reducing/oxidizing gases (H_2_/NO), the device’s conductivity can be linearly modulated (linearity factor 1.02/1.31), mimicking synaptic enhancement/inhibition and achieving 92.76% accuracy in MNIST pattern recognition. This approach paves the way for bio-inspired olfactory systems that integrate sensing and computing.

**Figure 12:**
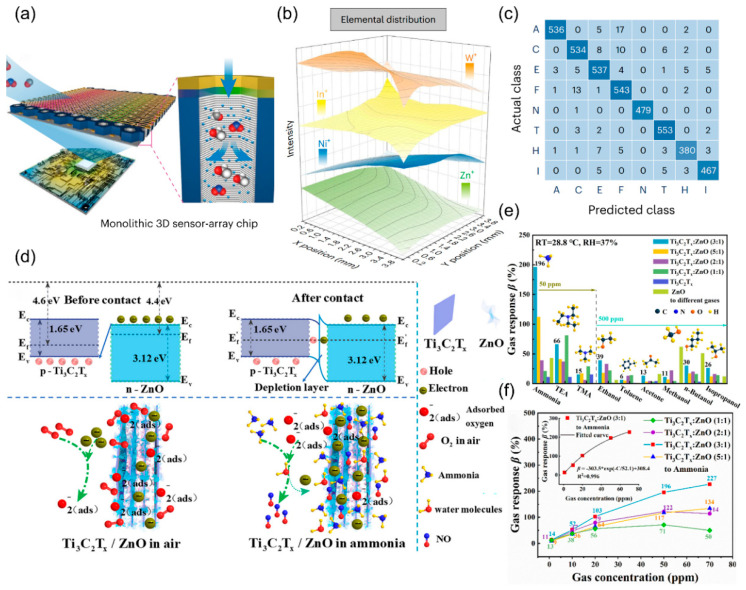
(**a**) Structure of a monolithic BOC with the correlated circuit. (**b**) Spatial distribution of elements in the MCI layer visualized using the loci of ToF-SIMS depth profiles. (**c**) Confusion matrix of the actual class and predicted class when recognizing eight gases (A, acetone; C, carbon monoxide; E, ethanol; F, formaldehyde; N, nitrogen dioxide; T, toluene; H, hydrogen; I, isobutylene). Reproduced with permission from ref. [115]. Copyright 2024 Springer Nature. (**d**) Sensing mechanism of Ti_3_C_2_T_x_/ZnO (3:1) sensor. (**e**) Comparison of Ti_3_C_2_T_x_ MXene, ZnO, and Ti_3_C_2_T_x_/ZnO sensors’ response to ammonia, triethylamine, trimethylamine gases with the concentration of 50 ppm, and to ethanol, toluene, acetone, methanol, n-butanol, and isopropanol gases with the concentration of 500 ppm at a low operating temperature (28 °C). (**f**) the line chart of gas concentration to the response and the inset is an index-related curve of Ti_3_C_2_T_x_/ZnO (3:1) about the correlation of gas concentration and response value. Reproduced with permission from ref. [118]. Copyright 2023 AIP Publishing.

On this basis, Wang et al. [115] further developed a single-chip bionic olfactory system (BOC) to overcome sensor diversity limitations through large-scale monolithic integration. This work employs vertically aligned PdO/SnO_2_ nanotube arrays supported by porous alumina templates and combines atomic layer deposition (ALD) and mask-assisted sputtering techniques to construct a two-dimensional multi-metal oxide interface layer (ZnO/NiO/In_2_O_3_/WO_3_) on the sensing layer. This enables a single chip to accommodate 10,000 independently addressable sensor arrays in Figure 12a. Through elemental gradient design (Figure 12b), the sensor array achieves spatial variation in material composition, mimicking the diversity of biological olfactory receptors. Using a convolutional neural network, the chip accurately classifies eight gases (99.04%), analyzes mixed gases (maximum 8.12% error), and distinguishes 24 complex odors (Figure 12c). It also integrates with a robot dog’s visual sensors for blind object recognition. These studies highlight the advantages of biomimetic strategies, advancing the development of chemiresistive sensors toward high discriminative and low-power integrated systems.

Additionally, creating material heterojunctions is an effective way to improve sensor selectivity for specific gases [116,117]. Yao et al. [118] developed a Ti_3_C_2_T_x_/ZnO p-n heterojunction using a hydrothermal method to enhance room-temperature ammonia detection. By combining p-type MXene (Ti_3_C_2_T_x_) with n-type ZnO at a 3:1 mass ratio, they achieved a sensor response of 196% for 50 ppm NH_3_ at 28 °C, higher than that of Ti_3_C_2_T_x_-based gas sensors (~2%) (Figure 12d). This is the first successful use of MXene/metal oxide heterojunctions for selective ammonia sensing, offering a new design approach for low-power sensing, as shown in Figure 12e,f.

### 4.2. Artificial Intelligence-Assisted Sensing

**Figure 13:**
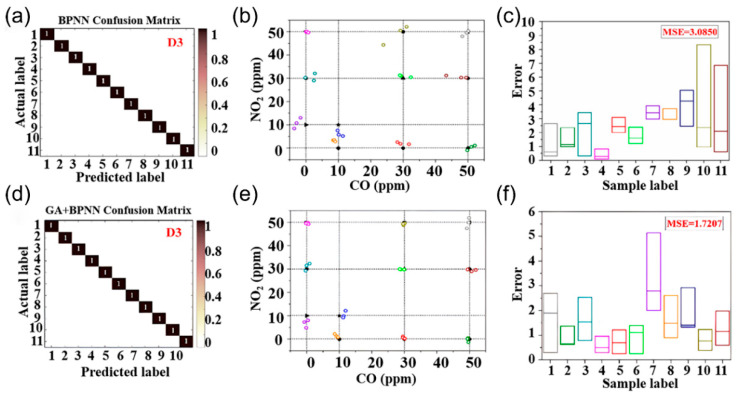
(**a**–**c**) Corresponds to the normalized confusion matrix, correlation performance, and predicted concentration box diagram of BPNN detection of CO and NO_2_. (**d**–**f**) Corresponds to the normalized confusion matrix of GA plus BPNN for detecting CO and NO_2_, the correlation performance, and the predicted concentration box diagram. Reproduced with permission from ref. [125]. Copyright 2021 Elsevier.

Artificial intelligence (AI) is revolutionizing sensor signal analysis and decision-making by using machine learning and deep learning to process sensor data and tackle cross-sensitivity. Techniques like linear discriminant analysis (LDA) [119], partial least squares regression (PLSR) [120], support vector machines (SVM) [121], principal component analysis (PCA) [122], parallel neural networks (PNN) [123], and convolutional neural networks (CNN) [124] are applied to chemiresistive sensor arrays for gas signal processing. This AI-sensor integration offers innovative approaches to enhance the selectivity of sensors. Chu et al. [125] developed a sensor array with four metal oxide semiconductor sensors and demonstrated that a GA-optimized BPNN algorithm significantly improved quantitative gas identification. Figure 13a,d show the normalized confusion matrices of BPNN and GA+BPNN, achieving 100% classification accuracy in the quantitative detection of CO and NO_2_ mixed gases, but the mean squared error (MSE) of BPNN was 3.085 (Figure 13b,c). In contrast, the MSE of the GA+BPNN optimized by the genetic algorithm was reduced to 1.7207, as shown in Figure 13e,f. This optimization by the genetic algorithm enhanced the initial weights and bias parameters of the BPNN, improving detection accuracy. The study integrates multi-sensor data fusion with deep learning to create a framework for selective gas detection in complex environments.

The AI model effectively learns gas “fingerprints”, ensuring stable identification despite sensor drift or environmental changes. Pan et al. [126] developed the HATN-DA algorithm to tackle drift and batch variation in mixed gas detection. This model combines multi-head Transformers for temporal dependencies, channel attention for sensor optimization, and capsule networks to output gas types and concentrations. By introducing domain adversarial learning based on Wasserstein distance, it compensates for drift without target domain labels, achieving 98.79% transfer accuracy on public datasets and reducing ethanol/methane/ethylene prediction errors to 3.32%/3.25%/3.01%. This approach enhances system robustness, overcoming traditional gas sensor limitations and providing dynamic adaptability in complex environments.

### 4.3. Multimodal Integrated System

**Figure 14:**
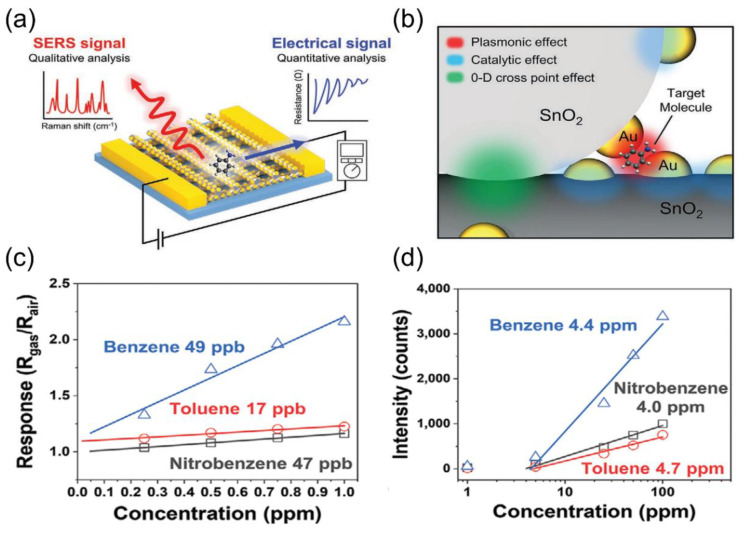
(**a**) Schematic illustration of detecting gas molecules and simultaneously collecting electrical signals for quantitative analysis and SERS signals for selective identification. (**b**) Schematic of the 3D-CMA structure depicting a cross-point junction of Au nanoparticle-decorated SnO_2_ nanowires. Calculated LOD of (**c**) electrical sensing and (**d**) optical sensing for each target gas. Reproduced with permission from ref. [128]. Copyright 2021 John Wiley and Sons.

To address the limitations of single-sensor systems, multimodal integrated systems combining various sensor types and technologies have become essential for improving selectivity and reliability. Li et al. [127] combined data from an electronic nose, an electronic tongue, and a colorimeter with machine learning algorithms to predict the freshness of frozen mackerel. Using PAC, random forest regression, XGBoost, and artificial neural networks, the multi-sensor fusion significantly enhanced the accuracy of predicting lipid and protein oxidation, proving the effectiveness of the “data fusion + machine learning” approach. In addition, the system can significantly improve the selectivity and quantitative analysis capabilities of gas detection by integrating complementary sensing technologies. Yeon Sik Jung’s team developed a multimodal gas sensor using 3D cross-multifunctional nanostructures (3D-CMA) [128]. This sensor combines chemiresistive sensing and surface-enhanced Raman spectroscopy (SERS) to selectively and quantitatively detect gases with similar molecular structures in Figure 14a, such as nitrobenzene and toluene. The structure is constructed via solvent-assisted nano-transfer printing, forming quantum contacts at SnO_2_ nanowire intersections to boost electrical sensitivity and using nano-gaps with Au NP modification as SERS hotspots (Figure 14b). This enables the sensor to provide both electrical signals and SERS fingerprint spectra for quantitative and qualitative analysis, with detection limits of <50 ppb and <5 ppm, respectively, as shown in Figure 14c,d. This design overcomes the bottlenecks of traditional sensors in selectivity, sensitivity, and mixed gas analysis, providing a new platform for environmental monitoring and biomedical diagnostics.

Furthermore, Burgués et al. [129] proposed the multimodal system for dynamic spatial monitoring by developing SNIFFDRONE, a drone-mounted chemical sensor system with 21 gas sensors and a 10 m sampling tube to minimize rotor wash interference. This system allows real-time odor concentration measurement at wastewater treatment plants. They implemented a dynamic calibration strategy, collecting transient sensor signals and odor samples according to EN13725 standards during drone hovering. Using PLSA, they created a model linking sensor responses to actual odor concentrations measured by dynamic olfactometry. This approach reduces prediction errors by 33%, increases the correlation coefficient with olfactometry to 0.86, and establishes 95% consistency limits at [0.25×, 3.91×]. The system was the first to spatially map odor concentrations in WWTPs, identifying major hotspots through 225 aerial survey points in a 200 × 100 m^2^ area, demonstrating the practical value of multimodal sensing in complex environments. The multimodal integrated system evolved from enhancing classification and accuracy in the lab through multi-sensor fusion to achieving spatial monitoring outdoors with a drone-mounted setup. A dynamic calibration strategy has effectively addressed the mismatch between transient field signals and steady-state laboratory data.

## 5. Conclusions and Future Perspective

In this review, we focus on the strategies reported in the literature for enhancing the selectivity of various gas-sensitive chemiresistive materials, addressing a persistent challenge through material innovation and system engineering.

Significant advancements have been made in improving the selectivity of chemiresistive gas sensors through various material modification strategies. Key approaches include (i) nanostructuring and morphology control of semiconductor metal oxides (SMOs) to increase surface area and active sites; (ii) functionalization with noble metal catalysts (e.g., Pd, Pt) or dopants to enhance specific gas-catalyst interactions and promote catalytic reactions; (iii) defect engineering (e.g., oxygen vacancy manipulation) to tailor surface chemistry and charge transfer processes; (iv) construction of heterojunctions (e.g., p-n junctions, Schottky barriers) to modulate electron transport and energy barriers; and (v) development of hybrid materials such as phosphorene, conductive MOFs, COFs, and composites like rGO-Ru OEP, which offer tunable electronic properties and selective gas permeation.

Additionally, surface modification with organic ligands and the integration of molecular sieving or filter layers have shown promise in enhancing specificity. Despite the aforementioned enhancements, numerous challenges continue to exist. Many strategies suffer from poor reproducibility due to difficulties in controlling catalyst size/distribution, sensitivity to environmental conditions (e.g., humidity), and limited long-term stability. The selectivity mechanisms are often not fully understood at the atomic level, hindering the development of universal design principles. Future efforts must focus on combining advanced in situ characterization techniques (e.g., operando XRD, XPS) and computational modeling (DFT) to decouple the dynamics of gas adsorption, surface reactions, and charge transfer. This will help establish clearer structure–activity relationships. Moreover, for practical applications, a systems-level approach is essential. This includes integrating selective materials with sensor arrays, microfluidic pre-concentration or separation columns, and artificial intelligence (AI)/machine learning algorithms for pattern recognition to handle complex gas mixtures and mitigate cross-sensitivity. Addressing issues like power consumption, manufacturing scalability, and robustness in real-world conditions (e.g., variable humidity/temperature) is crucial for reliable deployment in areas such as medical diagnostics, environmental monitoring, and industrial safety. Ultimately, the goal is to develop adaptive, multifunctional sensing platforms that combine material innovation with intelligent systems for high-fidelity and reliable gas discrimination.

In future research, it is imperative to prioritize the development of unified evaluation frameworks that rigorously assess sensor performance under practical conditions, including encompassing complex gas mixtures and defined humidity ranges. Material innovation should focus on creating multifunctional systems that integrate self-healing biomimetic coatings with stimuli-responsive layers to mitigate drift and environmental interference. Advanced manufacturing techniques, such as spark ablation printing (VSP-P1), could facilitate the high-throughput fabrication of precisely engineered multi-component sensing films, thereby accelerating the discovery of optimal materials. At the system architecture level, incorporating AI-driven dynamic baseline correction within miniaturized “lab-on-a-chip” platforms which include micro-preconcentrators, chromatographic columns, and optimized sensor arrays-holds the potential to enhance specificity while reducing power consumption. Additionally, biomimetic designs that mimic the spatial patterning of olfactory receptors may further refine array topology for complex odor discrimination. In conclusion, the integration of these material, structural, and systemic strategies will facilitate advancements in noninvasive medical diagnostics such as breath-based disease detection and real-time environmental monitoring, bridging the critical selectivity-reliability gap for ubiquitous sensor deployment.

## Figures and Tables

**Table 1:** Recently reports of different modification methods on the gas-sensing performance of chemiresistive gas sensors.

Modification Method	Materials	Target Gas	Temperature (°C)	Interferents	Response/Conc (ppm)	Ref.
Noble Metal Loading	Pt/ZnO	H_2_S	310	VOCs and NH_3_	118 ^a^/5.5	[101]
Noble Metal Loading	Pd/SnO_2_	Benzene	RT	benzene, m-xylene, toluene, acetone, acetaldehyde, isoprene, methanol, ethanol, CO, hdrogen, and ethylbenzene	2.1 ^a^/1	[102]
Nanostructure Engineering	Au/WO_3_	Isoprene	275	not investigated	11.3 ^a^/0.1	[103]
Nanostructure Engineering	Ag/SnO_2_	TEA	170	not investigated	1700 ^a^/100	[104]
Bimetallic Core–Shell Loading	PdAu/W_18_O_49_	H_2_S	100	CH_4_	55.5 ^a^/50	[105]
Bimetallic Noble Metal Loading	AgPd/In_2_O_3_	Toluene	180	Methanol, ammonia, acetone, ethanol and formaldehyde	15.9 ^a^/1	[106]
Metal Oxide Loading	CuO_x_/Co_3_O_4_	formaldehyde	75	acetone, toluene, ethanol, NO, acetaldehyde, NH_3_, CO, CH_4_	5.2% ^c^/1	[107]
Noble Metal Decoration	Pd hemitubes	H_2_	RT	NH_3_, CO, NO_2_, and H_2_S	2.1% ^e^/1	[108]
Pd-Doping	WO_3_ nanomaterials	Acetone	175	Acetone, Ethanol, Ammonium hydroxide, Methyl cyanide, Dichloromethane, Trichloromethane, Ether, Acetic acid and Hexane	150% ^a^/50	[109]
Photoelectronic Sensing	MoS_2_ with led light	NO_2_	RT	not investigated	12% ^e^/200	[110]
Nanostructure Engineering	3D epitaxial graphene nanowalls	O_2_	RT	N_2_ and H_2_	77% ^d^/500	[111]
Heterojunction Construction	5CeO_2_/Co_3_O_4_	CO	200	not investigated	184% ^b^/50	[112]
Heterojunction Construction	Co_3_O_4_/ZnO	CO	210	H_2_, CH_4_ and NH_3_	38% ^c^/10	[113]

^a^: R_g_/R_a_. ^b^: (R_g_ − R_a_)/R_a_. ^c^: R_a_/R_g_ × 100%. ^d^: ∆I/I_0_. ^e^: (∆R/R_0_) × 100%.

## Data Availability

The data that has been used is confidential.

## References

[1] Chaudhary V., Taha B.A., Lucky S., Rustagi S., Khosla A., Papakonstantinou P., Bhalla N. (2024). Nose-on-Chip Nanobiosensors for Early Detection of Lung Cancer Breath Biomarkers. ACS Sens..

[2] Baharfar M., Lin J., Kilani M., Zhao L., Zhang Q., Mao G. (2023). Gas nanosensors for health and safety applications in mining. Nanoscale Adv..

[3] Shaalan N.M., Ahmed F., Saber O., Kumar S. (2022). Gases in Food Production and Monitoring: Recent Advances in Target Chemiresistive Gas Sensors. Chemosensors.

[4] Milone A., Monteduro A.G., Rizzato S., Leo A., Di Natale C., Kim S.S., Maruccio G. (2023). Advances in Materials and Technologies for Gas Sensing from Environmental and Food Monitoring to Breath Analysis. Adv. Sustain. Syst..

[5] Srinivasan P., Ezhilan M., Kulandaisamy A.J., Babu K.J., Rayappan J.B.B. (2019). Room temperature chemiresistive gas sensors: Challenges and strategies-a mini review. J. Mater. Sci. Mater. Electron..

[6] Bakker E., Telting-Diaz M. (2002). Electrochemical sensors. Anal. Chem..

[7] Zong B., Wu S., Yang Y., Li Q., Tao T., Mao S. (2025). Smart Gas Sensors: Recent Developments and Future Prospective. Nano-Micro Lett..

[8] Li P., Li J., Song S., Chen J., Zhong N., Xie Q., Liu Y., Wan B., He Y., Karimi-Maleh H. (2025). Recent advances in optical gas sensors for carbon dioxide detection. Measurement.

[9] Hyodo T., Shimizu Y. (2020). Adsorption/Combustion-type Micro Gas Sensors: Typical VOC-sensing Properties and Material-design Approach for Highly Sensitive and Selective VOC Detection. Anal. Sci..

[10] Naganaboina V.R., Singh S.G. (2023). Chemiresistive gas sensors From novel gas-sensing materials to electrode structure. Chem. Phys. Rev..

[11] Reddy B.K.S., Borse P.H. (2021). Review—Recent Material Advances and Their Mechanistic Approaches for Room Temperature Chemiresistive Gas Sensors. J. Electrochem. Soc..

[12] Turlybekuly A., Shynybekov Y., Soltabayev B., Yergaliuly G., Mentbayeva A. (2024). The Cross-Sensitivity of Chemiresistive Gas Sensors: Nature, Methods, and Peculiarities: A Systematic Review. ACS Sens..

[13] Zhu L.-Y., Ou L.-X., Mao L.-W., Wu X.-Y., Liu Y.-P., Lu H.-L. (2023). Advances in Noble Metal-Decorated Metal Oxide Nanomaterials for Chemiresistive Gas Sensors: Overview. Nano-Micro Lett..

[14] Kumar A., Mazumder J.T., Joyen K., Favier F., Mirzaei A., Kim J.-Y., Kwoka M., Bechelany M., Jha R.K., Kumar M. (2025). Defect engineering approaches for metal oxide semiconductor-based chemiresistive gas sensing. Coord. Chem. Rev..

[15] Jian Y., Hu W., Zhao Z., Cheng P., Haick H., Yao M., Wu W. (2020). Gas Sensors Based on Chemi-Resistive Hybrid Functional Nanomaterials. Nano-Micro Lett..

[16] Yang B., Myung N.V., Tran T.T. (2021). 1D Metal Oxide Semiconductor Materials for Chemiresistive Gas Sensors: A Review. Adv. Electron. Mater..

[17] Lee H.S., Kim J., Moon H., Lee W. (2021). Hydrogen Gas Sensors Using Palladium Nanogaps on an Elastomeric Substrate. Adv. Mater..

[18] Tang Y.M., Ong C.W. (2011). Analysis and improvement of cyclic stability of H_2_ sensing properties of Pd/Mg–Ni films. Int. J. Hydrogen Energy.

[19] Wang Q., Wang M., Zheng K., Ye W., Zhang S., Wang B., Long X. (2024). High-Performance Room Temperature Ammonia Sensors Based on Pure Organic Molecules Featuring B-N Covalent Bond. Adv. Sci..

[20] Alzate-Carvajal N., Luican-Mayer A. (2020). Functionalized Graphene Surfaces for Selective Gas Sensing. ACS Omega.

[21] (2023). Chemiresistive sensing with functionalized carbon nanotubes. Nat. Rev. Methods Prim..

[22] Drozdowska K., Smulko J. (2025). Selective light-activation of sensing regions in hybrid Au-graphene-TiO_2_ chemiresistive gas sensor. Sens. Actuators B Chem..

[23] Mei H., Peng J., Wang T., Zhou T., Zhao H., Zhang T., Yang Z. (2024). Overcoming the Limits of Cross-Sensitivity: Pattern Recognition Methods for Chemiresistive Gas Sensor Array. Nano-Micro Lett..

[24] Dong W., Zhao J., Hu R., Dong Y., Tan L. (2017). Differentiation of Chinese robusta coffees according to species, using a combined electronic nose and tongue, with the aid of chemometrics. Food Chem..

[25] Bulemo P.M., Kim D.-H., Shin H., Cho H.-J., Koo W.-T., Choi S.-J., Park C., Ahn J., Guntner A.T., Penner R.M. (2025). Selectivity in Chemiresistive Gas Sensors: Strategies and Challenges. Chem. Rev..

[26] Galvani M., Freddi S., Sangaletti L. (2024). Disclosing Fast Detection Opportunities with Nanostructured Chemiresistor Gas Sensors Based on Metal Oxides, Carbon, and Transition Metal Dichalcogenides. Sensors.

[27] Yang J., Sun R., Bao X., Liu J., Ng J.W., Tang B., Liu Z. (2025). Enhancing Selectivity of Two-Dimensional Materials-Based Gas Sensors. Adv. Funct. Mater..

[28] Yamazoe N., Shimanoe K. (2011). Theoretical approach to the gas response of oxide semiconductor film devices under control of gas diffusion and reaction effects. Sens. Actuators B Chem..

[29] Majhi S.M., Navale S.T., Mirzaei A., Kim H.W., Kim S.S. (2023). Strategies to boost chemiresistive sensing performance of In_2_O_3_-based gas sensors: An overview. Inorg. Chem. Front..

[30] Franco M.A., Conti P.P., Andre R.S., Correa D.S. (2022). A review on chemiresistive ZnO gas sensors. Sens. Actuators Rep..

[31] Jeong S.Y., Kim J.S., Lee J.H. (2020). Rational Design of Semiconductor-Based Chemiresistors and their Libraries for Next-Generation Artificial Olfaction. Adv. Mater..

[32] Ding Y., Guo X., Zhou Y., He Y., Zang Z. (2022). Copper-based metal oxides for chemiresistive gas sensors. J. Mater. Chem. C.

[33] Wusiman M., Taghipour F. (2021). Methods and mechanisms of gas sensor selectivity. Crit. Rev. Solid State Mater. Sci..

[34] Kim Y., Sohn I., Shin D., Yoo J., Lee S., Yoon H., Park J., Chung S.-m., Kim H. (2024). Recent Advances in Functionalization and Hybridization of Two-Dimensional Transition Metal Dichalcogenide for Gas Sensor. Adv. Eng. Mater..

[35] Ghosh R., Aslam M., Kalita H. (2022). Graphene derivatives for chemiresistive gas sensors: A review. Mater. Today Commun..

[36] Recum P., Hirsch T. (2024). Graphene-based chemiresistive gas sensors. Nanoscale Adv..

[37] Kumar S., Mirzaei A., Kumar A., Lee M.H., Ghahremani Z., Kim T.-U., Kim J.Y., Kwoka M., Kumar M., Kim S.S. (2024). Nanoparticles anchored strategy to develop 2D MoS_2_ and MoSe_2_ based room temperature chemiresistive gas sensors. Coord. Chem. Rev..

[38] Choi S.-J., Kim I.-D. (2018). Recent Developments in 2D Nanomaterials for Chemiresistive-Type Gas Sensors. Electron. Mater. Lett..

[39] Srinivasan P., Samanta S., Krishnakumar A., Rayappan J.B.B., Kailasam K. (2021). Insights into g-C_3_N_4_ as a chemi-resistive gas sensor for VOCs and humidity—A review of the state of the art and recent advancements. J. Mater. Chem. A.

[40] Joshi N., Hayasaka T., Liu Y., Liu H., Oliveira O.N., Lin L. (2018). A review on chemiresistive room temperature gas sensors based on metal oxide nanostructures, graphene and 2D transition metal dichalcogenides. Microchim. Acta.

[41] Balamurugan C., Song S.-J., Kim H.-S. (2018). Enhancing Gas Response Characteristics of Mixed Metal Oxide Gas Sensors. J. Korean Ceram. Soc..

[42] Bulemo P.M., Cho H.-J., Kim D.-H., Kim I.-D. (2018). Facile Synthesis of Pt-Functionalized Meso/Macroporous SnO2 Hollow Spheres through in Situ Templating with SiO_2_ for H_2_S Sensors. ACS Appl. Mater. Interfaces.

[43] Cho H.-J., Kim S.-J., Choi S.-J., Jang J.-S., Kim I.-D. (2017). Facile synthetic method of catalyst-loaded ZnO nanofibers composite sensor arrays using bio-inspired protein cages for pattern recognition of exhaled breath. Sens. Actuators B Chem..

[44] Jeong Y.J., Koo W.-T., Jang J.-S., Kim D.-H., Cho H.-J., Kim I.-D. (2018). Chitosan-templated Pt nanocatalyst loaded mesoporous SnO_2_ nanofibers: A superior chemiresistor toward acetone molecules. Nanoscale.

[45] Degler D., Weimar U., Barsan N. (2019). Current Understanding of the Fundamental Mechanisms of Doped and Loaded Semiconducting Metal-Oxide-Based Gas Sensing Materials. ACS Sens..

[46] Chen Z., Yuan P., Chen C., Wang X., Wang J., Jia J., Davaasuren B., Lai Z., Khashab N.M., Huang K.-W. (2024). Balancing Pd-H Interactions: Thiolate-Protected Palladium Nanoclusters for Robust and Rapid Hydrogen Gas Sensing. Adv. Mater..

[47] Shringi A.K., Kumar A., Das M., Kim S.S., Kim H.W., Kumar M. (2023). Ag catalysts boosted NO_2_ gas sensing performance of RF sputtered α-Fe2O3 films. Sens. Actuators B Chem..

[48] Bae G., Kim M., Lee A., Ji S., Jang M., Yim S., Song W., Lee S.S., Yoon D.H., An K.-S. (2022). Nanometric lamination of zinc oxide nanofilms with gold nanoparticles for self-perceived periodontal disease sensors. Compos. Part B Eng..

[49] Teng L., Liu Y., Ikram M., Liu Z., Ullah M., Ma L., Zhang X., Wu H., Li L., Shi K. (2019). One-step synthesis of palladium oxide-functionalized tin dioxide nanotubes: Characterization and high nitrogen dioxide gas sensing performance at room temperature. J. Colloid Interface Sci..

[50] Hassan K., Chung G.-S. (2017). Catalytically activated quantum-size Pt/Pd bimetallic core–shell nanoparticles decorated on ZnO nanorod clusters for accelerated hydrogen gas detection. Sens. Actuators B Chem..

[51] Liu B., Li K., Luo Y., Gao L., Duan G. (2021). Sulfur spillover driven by charge transfer between AuPd alloys and SnO_2_ allows high selectivity for dimethyl disulfide gas sensing. Chem. Eng. J..

[52] Le H.-J., Van Dao D., Yu Y.-T. (2020). Superfast and efficient hydrogen gas sensor using PdAu_alloy_@ ZnO core–shell nanoparticles. J. Mater. Chem. A.

[53] Li G., Wang X., Yan L., Wang Y., Zhang Z., Xu J. (2019). PdPt Bimetal-Functionalized SnO_2_ Nanosheets: Controllable Synthesis and its Dual Selectivity for Detection of Carbon Monoxide and Methane. ACS Appl. Mater. Interfaces.

[54] Dvorak F., Camellone M.F., Tovt A., Nguyen-Dung T., Negreiros F.R., Vorokhta M., Skala T., Matolinova I., Myslivecek J., Matolin V. (2016). Creating single-atom Pt-ceria catalysts by surface step decoration. Nat. Commun..

[55] Xu Y., Zheng W., Liu X., Zhang L., Zheng L., Yang C., Pinna N., Zhang J. (2020). Platinum single atoms on tin oxide ultrathin films for extremely sensitive gas detection. Mater. Horiz..

[56] Zhao H., Ge W., Li X., Zhao T., Luo Z., Wang R., Wang S., Shang S., Zhang Q., DiWu H. (2023). High NO_2_ sensing for room temperature: A multifunctional hydrophobic sensitive layer with compatible flexible and ultraviolet activated. Chem. Eng. J..

[57] Zhang B., Xia Y., Zhang S., Xu Y., Dong Y., Yu P., Ni Y., Wei Q., Guo L., Wang J. (2022). ZnO Nanowires with Increasing Aspect Ratios for Room-Temperature NO_2_ Gas Sensing. ACS Appl. Nano Mater..

[58] Srinivasan P., Rayappan J.B.B. (2018). Growth of Eshelby twisted ZnO nanowires through nanoflakes & nanoflowers: A room temperature ammonia sensor. Sens. Actuators B Chem..

[59] Wang T., Xing Q., Zhai R., Huang T., Song P. (2024). Defect Engineering for SnO_2_ Improves NO_2_ Gas Sensitivity by Plasma Spraying. ACS Sens..

[60] Li Z., Liu X., Zhou M., Zhang S., Cao S., Lei G., Lou C., Zhang J. (2021). Plasma-induced oxygen vacancies enabled ultrathin ZnO films for highly sensitive detection of triethylamine. J. Hazard. Mater..

[61] Shin K.Y., Mirzaei A., Oum W., Yu D.J., Kang S., Kim E.B., Kim H.M., Kim S.S., Kim H.W. (2022). Enhancement of selective NO_2_ gas sensing via Xenon ion irradiation of ZnO nanoparticles. Sens. Actuators B Chem..

[62] Paolucci V., De Santis J., Ricci V., Lozzi L., Giorgi G., Cantalini C. (2022). Bidimensional Engineered Amorphous a-SnO_2_ Interfaces: Synthesis and Gas Sensing Response to H2S and Humidity. ACS Sens..

[63] Chang C.M., Hon M.H., Leu I.C. (2010). Preparation of ZnO nanorod arrays with tailored defect-related characterisitcs and their effect on the ethanol gas sensing performance. Sens. Actuators B Chem..

[64] Shen J.-Y., Wang M.-D., Wang Y.-F., Hu J.-Y., Zhu Y., Zhang Y.X., Li Z.-J., Yao H.-C. (2018). Iron and carbon codoped WO_3_ with hierarchical walnut-like microstructure for highly sensitive and selective acetone sensor. Sens. Actuators B Chem..

[65] Li Q., Wang L., Xiao A., Zhu L., Yang Z. (2025). Hydrogen sensing towards palladium-based nanocomposites: A review. Int. J. Hydrogen Energy.

[66] Tanaka T., Hoshino S., Takahashi T., Uchida K. (2018). Nanoscale Pt thin film sensor for accurate detection of ppm level hydrogen in air at high humidity. Sens. Actuators B Chem..

[67] Alkhabet M.M., Girei S.H., Al-Isawi Z.K., Shareef O.S.F., Farhan A.H., Altalebi O., Khalaf A.L., Jaafar J.A., Yaacob M.H. (2025). Palladium (Pd) coated fiber optic hydrogen sensors: A review. Mater. Sci. Semicond. Process..

[68] Ramgir N.S., Yang Y., Zacharias M. (2010). Nanowire-based sensors. Small.

[69] Ding M., Liu Y., Wang G., Zhao Z., Yin A., He Q., Huang Y., Duan X. (2016). Highly Sensitive Chemical Detection with Tunable Sensitivity and Selectivity from Ultrathin Platinum Nanowires. Small.

[70] Koo W.-T., Qiao S., Ogata A.F., Jha G., Jang J.-S., Chen V.T., Kim I.-D., Penner R.M. (2017). Accelerating Palladium Nanowire H_2_ Sensors Using Engineered Nanofiltration. ACS Nano.

[71] Gautam Y.K., Sanger A., Kumar A., Chandra R. (2015). A room temperature hydrogen sensor based on Pd-Mg alloy and multilayers prepared by magnetron sputtering. Int. J. Hydrogen Energy.

[72] Hassan K., Chung G.-S. (2017). Fast and reversible hydrogen sensing properties of Pd-capped Mg ultra-thin films modified by hydrophobic alumina substrates. Sens. Actuators B Chem..

[73] Wang B., Zhu Y., Chen Y., Song H., Huang P., Dzung Viet D. (2017). Hydrogen sensor based on palladium-yttrium alloy nanosheet. Mater. Chem. Phys..

[74] Kim D.-H., Kim S.-J., Shin H., Koo W.-T., Jang J.-S., Kang J.-Y., Jeong Y.J., Kim I.-D. (2019). High-Resolution, Fast, and Shape-Conformable Hydrogen Sensor Platform: Polymer Nanofiber Yarn Coupled with Nanograined Pd@Pt. ACS Nano.

[75] Yang G.G., Kim D.-H., Samal S., Choi J., Roh H., Cunin C.E., Lee H.M., Kim S.O., Dinca M., Gumyusenge A. (2023). Polymer-Based Thermally Stable Chemiresistive Sensor for Real-Time Monitoring of NO_2_ Gas Emission. ACS Sens..

[76] Ghoorchian A., Alizadeh N. (2018). Chemiresistor gas sensor based on sulfonated dye-doped modified conducting polypyrrole film for high sensitive detection of 2,4,6-trinitrotoluene in air. Sens. Actuators B Chem..

[77] Song W., Sun J., Wang Q., Wu H., Zheng K., Wang B., Wang Z., Long X. (2024). n-Type boron β-diketone-containing conjugated polymers for high-performance room temperature ammonia sensors. Mater. Horiz..

[78] Ngai J.H.L., Gao X., Kumar P., Polena J., Li Y. (2021). A Highly Stable Diketopyrrolopyrrole (DPP) Polymer for Chemiresistive Sensors. Adv. Electron. Mater..

[79] Rath R.J., Naficy S., Giaretta J., Oveissi F., Yun J., Dehghani F., Farajikhah S. (2024). Chemiresistive Sensor for Enhanced CO_2_ Gas Monitoring. ACS Sens..

[80] Rath R.J., Farajikhah S., Oveissi F., Shahrbabaki Z., Yun J., Naficy S., Dehghani F. (2023). A Polymer-Based Chemiresistive Gas Sensor for Selective Detection of Ammonia Gas. Adv. Sens. Res..

[81] He W., Li Q.-W., Chen S., Liu H., Cheng Z., Li S., Lyu W., Xu G., Chen Y.-J., Liao Y. (2025). Enhanced Conductivity in Conjugated Microporous Polymers via Integrating of Carbon Nanotubes for Ultrasensitive NO_2_ Chemiresistive Sensor. Small.

[82] Thangamani G.J., Deshmukh K., Nambiraj N.A., Pasha S.K.K. (2021). Chemiresistive gas sensors based on vanadium pentoxide reinforced polyvinyl alcohol/polypyrrole blend nanocomposites for room temperature LPG sensing. Synth. Met..

[83] Jo Y.K., Jeong S.-Y., Moon Y.K., Jo Y.-M., Yoon J.-W., Lee J.-H. (2021). Exclusive and ultrasensitive detection of formaldehyde at room temperature using a flexible and monolithic chemiresistive sensor. Nat. Commun..

[84] Huang C., Sun W., Jin Y., Guo Q., Muecke D., Chu X., Liao Z., Chandrasekhar N., Huang X., Lu Y. (2024). A General Synthesis of Nanostructured Conductive Metal-Organic Frameworks from Insulating MOF Precursors for Supercapacitors and Chemiresistive Sensors. Angew. Chem. Int. Ed. Engl..

[85] Ambreen S., Gupta D.K., Kumar H., Sharma A., Arun S., Kumar S., Saraswat A., Mishra A.K. (2025). Advancements in 2D TMDs as Sensors: From Materials to Real-World Applications. Luminescence.

[86] Zhang J., Liu L., Yang Y., Huang Q., Li D., Zeng D. (2021). A review on two-dimensional materials for chemiresistive- and FET-type gas sensors. Phys. Chem. Chem. Phys..

[87] Wadhwa R., Kumar A., Sarkar R., Mohanty P.P., Kumar D., Deswal S., Kumar P., Ahuja R., Chakraborty S., Kumar M. (2023). Pt Nanoparticles on Vertically Aligned Large-Area MoS2Flakes for Selective H_2_Sensing at Room Temperature. ACS Appl. Nano Mater..

[88] Bharathi P., Harish S., Mathankumar G., Krishna Mohan M., Archana J., Kamalakannan S., Prakash M., Shimomura M., Navaneethan M. (2022). Solution processed edge activated Ni-MoS_2_ nanosheets for highly sensitive room temperature NO_2_ gas sensor applications. Appl. Surf. Sci..

[89] Bharathi P., Harish S., Shimomura M., Ponnusamy S., Krishna Mohan M., Archana J., Navaneethan M. (2022). Conductometric NO_2_ gas sensor based on Co-incorporated MoS_2_ nanosheets for room temperature applications. Sens. Actuators B Chem..

[90] Tian X., Cui X., Xiao Y., Chen T., Xiao X., Wang Y. (2023). Pt/MoS_2_/Polyaniline Nanocomposite as a Highly Effective Room Temperature Flexible Gas Sensor for Ammonia Detection. ACS Appl. Mater. Interfaces.

[91] Li Z., Liao Y., Liu Y., Zeng W., Zhou Q. (2023). Room temperature detection of nitrogen dioxide gas sensor based on Pt-modified MoSe2 nanoflowers: Experimental and theoretical analysis. Appl. Surf. Sci..

[92] Niu Y., Zeng J., Liu X., Li J., Wang Q., Li H., Rooij N.F.d., Wang Y., Zhou G. (2021). A Photovoltaic Self-Powered Gas Sensor Based on All-Dry Transferred MoS_2_/GaSe Heterojunction for ppb-Level NO2 Sensing at Room Temperature. Adv. Sci..

[93] Naganaboina V.R., Singh S.G. (2021). Graphene-CeO_2_ based flexible gas sensor: Monitoring of low ppm CO gas with high selectivity at room temperature. Appl. Surf. Sci..

[94] Jagannathan M., Dhinasekaran D., Rajendran A.R., Subramaniam B. (2022). Selective room temperature ammonia gas sensor using nanostructured ZnO/CuO@graphene on paper substrate. Sens. Actuators B Chem..

[95] Emam S., Nasrollahpour M., Allen J.P., He Y., Hussein H., Shah H.S., Tavangarian F., Sun N.-X. (2022). A handheld electronic device with the potential to detect lung cancer biomarkers from exhaled breath. Biomed. Microdevices.

[96] Feicht P., Eigler S. (2018). Defects in Graphene Oxide as Structural Motifs. ChemNanoMat.

[97] Wang J., Singh B., Park J.-H., Rathi S., Lee I.-y., Maeng S., Joh H.-I., Lee C.-H., Kim G.-H. (2014). Dielectrophoresis of graphene oxide nanostructures for hydrogen gas sensor at room temperature. Sens. Actuators B Chem..

[98] Yang Z., Zhao L., Zhang Y., Xing Y., Wei Z., Xin C., Fei T., Liu S., Zhang T. (2023). Isolated Cu-N5 sites engineered polypyrrole-reduced graphene oxide hybrids for enhancing room-temperature DMMP sensing. Sens. Actuators B Chem..

[99] Thathsara T., Meilak J., Sangchap M., Harrison C., Hocking R., Shafiei M. (2023). Visible light active rGO nanosheet encapsulated Pd quantum-sized dots decorated TiO_2_ nano-spheres for hydrogen gas sensing at low temperatures. Int. J. Hydrogen Energy.

[100] Jayaramulu K., Esclance Dmello M., Kesavan K., Schneemann A., Otyepka M., Kment S., Narayana C., Kalidindi S.B., Varma R.S., Zboril R. (2021). A multifunctional covalently linked graphene–MOF hybrid as an effective chemiresistive gas sensor. J. Mater. Chem. A.

[101] Zhou Q., Xu L., Kan Z., Yang L., Chang Z., Dong B., Bai X., Lu G., Song H. (2022). A multi-platform sensor for selective and sensitive H_2_S monitoring: Three-dimensional macroporous ZnO encapsulated by MOFs with small Pt nanoparticles. J. Hazard. Mater..

[102] Weber I.C., Ruedi P., Sot P., Guntner A.T., Pratsinis S.E. (2022). Handheld Device for Selective Benzene Sensing over Toluene and Xylene. Adv. Sci..

[103] Park S.-W., Jeong S.-Y., Moon Y.K., Kim K., Yoon J.-W., Lee J.-H. (2022). Highly Selective and Sensitive Detection of Breath Isoprene by Tailored Gas Reforming: A Synergistic Combination of Macroporous WO_3_ Spheres and Au Catalysts. ACS Appl. Mater. Interfaces.

[104] Zhang J., Zhang B., Yao S., Li H., Chen C., Bala H., Zhang Z. (2022). Improved triethylamine sensing properties of fish-scale-like porous SnO_2_ nanosheets by decorating with Ag nanoparticles. J. Mater..

[105] Zhang W., Yuan T., Wang X., Xu J. (2022). Coal mine gases sensors with dual selectivity at variable temperatures based on a W_18_O_49_ ultra-fine nanowires/Pd@Au bimetallic nanoparticles composite. Sens. Actuators B Chem..

[106] Liu X., Duan X., Zhang C., Hou P., Xu X. (2022). Improvement toluene detection of gas sensors based on flower-like porous indium oxide nanosheets. J. Alloys Compd..

[107] D’Andria M., Krumeich F., Yao Z., Wang F.R., Guntner A.T. (2024). Structure-Function Relationship of Highly Reactive CuO_x_ Clusters on Co_3_O_4_ for Selective Formaldehyde Sensing at Low Temperatures. Adv. Sci..

[108] Cho M., Zhu J., Kim H., Kang K., Park I. (2019). Half-Pipe Palladium Nanotube-Based Hydrogen Sensor Using a Suspended Nanofiber Scaffold. ACS Appl. Mater. Interfaces.

[109] Li P., Zhang Z., Zhuang Z., Guo J., Fang Z., Fereja S.L., Chen W. (2021). Pd-Doping-Induced Oxygen Vacancies in One-Dimensional Tungsten Oxide Nanowires for Enhanced Acetone Gas Sensing. Anal. Chem..

[110] Tung P., Li G., Bekyarova E., Itkis M.E., Mulchandani A. (2019). MoS_2_-Based Optoelectronic Gas Sensor with Sub-parts-per-billion Limit of NO_2_ Gas Detection. ACS Nano.

[111] Roy P.K., Haider G., Chou T.-C., Chen K.-H., Chen L.-C., Chen Y.-F., Liang C.-T. (2019). Ultrasensitive Gas Sensors Based on Vertical Graphene Nanowalls/SiC/Si Heterostructure. ACS Sens..

[112] Qin C., Wei Z., Zhao X., Sun J., Cao J., Wang Y. (2025). Nanosheet-assembled hierarchical CeO_2_/Co_3_O_4_ heterostructures derived from bimetallic MOFs for enhanced CO detection. Sens. Actuators B Chem..

[113] Chen M., Li X., Li Y., Li Y., Qin Z., Wang Q. (2024). MOF-derived Co_3_O_4_ nanoparticles over direct grown ZnO nanoflower on ceramic for CO sensor with high selectivity. Sens. Actuators B Chem..

[114] Chun S.Y., Song Y.G., Kim J.E., Kwon J.U., Soh K., Kwon J.Y., Kang C., Yoon J.H. (2023). An Artificial Olfactory System Based on a Chemi-Memristive Device. Adv. Mater..

[115] Wang C., Chen Z., Chan C.L.J., Wan Z.a., Ye W., Tang W., Ma Z., Ren B., Zhang D., Song Z. (2024). Biomimetic olfactory chips based on large-scale monolithically integrated nanotube sensor arrays. Nat. Electron..

[116] Yu M., Li J., Yin D., Zhou Z., Wei C., Wang Y., Hao J. (2024). Enhanced oxygen anions generation on Bi2S3/Sb2S3 heterostructure by visible light for trace H_2_S detection at room temperature. J. Hazard. Mater..

[117] Cheng X., Yao Y., Zheng S., Wan Y., Wei C., Yang G., Yuan Y., Tsai H.-S., Wang Y., Hao J. (2025). Te@Se Core–Shell Heterostructures with Tunable Shell Thickness for Ultra-Stable NO_2_ Detection. ACS Sens..

[118] Yao L., Tian X., Cui X., Zhao R., Chen T., Xiao X., Wang Y. (2023). Low operating temperature and highly selective NH3 chemiresistive gas sensors based on a novel 2D Ti_3_C_2_T_x_/ZnO composite with p–n heterojunction. Appl. Phys. Rev..

[119] Zhu F., Gao J., Yang J., Ye N. (2022). Neighborhood linear discriminant analysis. Pattern Recognit..

[120] Ma H., Wang T., Li B., Cao W., Zeng M., Yang J., Su Y., Hu N., Zhou Z., Yang Z. (2022). A low-cost and efficient electronic nose system for quantification of multiple indoor air contaminants utilizing HC and PLSR. Sens. Actuators B Chem..

[121] Chauhan V.K., Dahiya K., Sharma A. (2019). Problem formulations and solvers in linear SVM: A review. Artif. Intell. Rev..

[122] Greenacre M., Groenen P.J.F., Hastie T., D’Enza A.L., Markos A., Tuzhilina E. (2022). Principal component analysis. Nat. Rev. Methods Primers.

[123] Sebastian A., Pannone A., Radhakrishnan S.S., Das S. (2019). Gaussian synapses for probabilistic neural networks. Nat. Commun..

[124] Gu J., Wang Z., Kuen J., Ma L., Shahroudy A., Shuai B., Liu T., Wang X., Wang G., Cai J. (2018). Recent advances in convolutional neural networks. Pattern Recognit..

[125] Chu J., Li W., Yang X., Wu Y., Wang D., Yang A., Yuan H., Wang X., Li Y., Rong M. (2021). Identification of gas mixtures via sensor array combining with neural networks. Sens. Actuators B Chem..

[126] Pan X., Chen J., Wen X., Hao J., Xu W., Ye W., Zhao X. (2023). A comprehensive gas recognition algorithm with label-free drift compensation based on domain adversarial network. Sens. Actuators B Chem..

[127] Li H., Wang Y., Zhang J., Li X., Wang J., Yi S., Zhu W., Xu Y., Li J. (2023). Prediction of the freshness of horse mackerel (*Trachurus japonicus*) using E-nose, E-tongue, and colorimeter based on biochemical indexes analyzed during frozen storage of whole fish. Food Chem..

[128] Han H.J., Cho S.H., Han S., Jang J.-S., Lee G.R., Cho E.N., Kim S.-J., Kim I.-D., Jang M.S., Tuller H.L. (2021). Synergistic Integration of Chemo-Resistive and SERS Sensing for Label-Free Multiplex Gas Detection. Adv. Mater..

[129] Burgués J., Doñate S., Esclapez M.D., Saúco L., Marco S. (2022). Characterization of odour emissions in a wastewater treatment plant using a drone-based chemical sensor system. Sci. Total. Environ..

